# Competition Between Liquid‐Liquid Crystalline Phase Separation (LLCPS) and Liquid‐Liquid Phase Separation (LLPS) in Amyloid Fibril Colloidal Systems

**DOI:** 10.1002/advs.202518781

**Published:** 2026-01-28

**Authors:** Milad Radiom, Raffaele Mezzenga

**Affiliations:** ^1^ Department of Health Sciences and Technology ETH Zürich Zürich 8092 Switzerland

**Keywords:** amyloid fibrils, condensates, liquid crystals, LLCPS, LLPS, phase diagram, phase transition

## Abstract

Amyloid fibrils are rigid, elongated protein aggregates that form condensed phases in functional biological assemblies and pathological deposits. From a colloidal physics perspective, two separation pathways can lead to these condensed phases: liquid–liquid phase separation (LLPS), which results from a trade‐off between entropy and enthalpy, and liquid–liquid crystalline phase separation (LLCPS), an essentially entropic process in which the condensed phase is a nematic liquid following precise symmetry rules. The interplay between these pathways in amyloid fibril dispersions is debated. Here we show that lysozyme and β‐lactoglobulin amyloid fibrils dominantly undergo LLCPS but exhibit a pH‐dependent transition to LLPS. Particularly, increasing pH lowers the critical concentration for formation of nematic condensates until, near isoelectric point, where condensates without a coherent nematic order form. This behavior is consistent with the classical picture of rigid rods retaining nematic order once formed, with enthalpic attraction overcoming entropic ordering at low charge densities. Our work establishes that LLCPS and LLPS in amyloid fibrils are separable and provides a framework for controlling fibril organization using simple solution conditions. The competition between LLCPS and LLPS may clarify the mesoscopic organization of amyloid fibrils in condensates in vivo and direct design principles of multiphase amyloid‐based materials.

## Graphical abstract

This study shows that amyloid fibrils form nematic condensates via liquid–liquid crystalline phase separation (LLCPS). With increasing pH, this transition shifts toward disordered condensates formed through liquid–liquid phase separation (LLPS), in which birefringence with lack of a coherent nematic field symmetry originates from enthalpic fibril interactions rather than entropy‐driven alignment. Therefore, solution conditions can regulate mesoscopic fibril organization through LLCPS–LLPS competition. This finding provides insights into amyloid organization in health and disease and informs design of multiphase amyloid‐based materials.

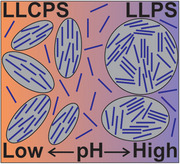

## 1 Introduction

Amyloid fibrils are highly ordered protein aggregates that arise from misfolding and self‐assembly of polypeptide chains into elongated filamentous structures, with a length‐to‐diameter ratio on the order of 10^1^–10^3^ [1]. They typically consist of one to several filaments, which are twisted together with a precise handedness into mature fibrils with a semiflexible to rigid structure [2]. A defining atomic feature in amyloid fibrils is a cross‐β strand structure, where hydrogen bonded β‐strands align perpendicularly to fibril axis, with interstrand spacing of 4.7 Å [1]. While amyloid fibrils have been traditionally associated with pathology in neurodegenerative and other disorders [3], they are increasingly recognized as functional elements across biological organisms [4]. Inspired by these properties, artificial amyloid fibrils, such as those derived from food proteins, are developed as building blocks for functional materials [5].

In living systems, amyloid fibrils are often found in soluble or in insoluble (deposit) assemblies, suggesting that phase separation acts as an organizing principle that may drive their biological outcome [6, 7]. Investigations with artificial fibrils have provided critical mechanistic insights into spatial organization of fibrils inside similar assemblies [8, 9]. Particularly, the structural and physical similarities between pathological, functional, and artificial amyloid fibrils—from molecular aggregation to microscopic organization [5]—provides an opportunity to bridge concepts from colloidal physics to the understanding of amyloid behavior in biology [7].

Two physical models are commonly utilized to describe phase transitions in colloidal systems [10]. The classical liquid–liquid phase separation (LLPS) partitions an isotropic solution of macromolecules into two isotropic liquids, one at dense (called condensate) and the other at dilute concentration boundaries. The liquid–liquid crystalline phase separation (LLCPS) couples this demixing with emergence of nematic order in the condensate, when macromolecules are anisotropic in shape or in pair‐interaction potential [8, 11]. The latter type of condensate, known as tactoid, exhibits anisotropic mechanical and transport properties [12], which are features that may play a role in functional amyloids. The transition from isotropic to isotropic‐nematic coexistence (LLCPS) is a generic entropic ordering phenomena in rod‐like macromolecular systems [13, 14].

Both LLPS and LLCPS can be dynamically tuned by parameters, such as pH, ionic strength, temperature, and crowding effects, providing a rich physical basis for the control of phase separations and fibril arrangements inside condensates [9, 11]. However, despite this intriguing possibility [15], and the potential relevance to amyloid organization in vivo, the competitive interplay between LLCPS and LLPS remains poorly understood.

To gain mechanistic insight into this interplay, we studied LLCPS and LLPS in solutions of model amyloid fibrils formed from chicken egg white lysozyme (also called hen egg white lysozyme, hereafter lysozyme) and β‐lactoglobulin. Both proteins are globular in native state; however, under acidic condition and elevated temperature, they undergo unfolding and self‐assembly to amyloid fibrils. Specifically, lysozyme is a cationic protein with a high content of arginine and lysine residues, resulting in fibrils that exhibit a dominantly positive surface charge below an isoelectric point near pH 10 [16, 17]. In contrast, β‐lactoglobulin is relatively richer in acidic residues, glutamate and aspartate, leading to fibrils with an isoelectric point near pH 5.0 [17, 18]. We focused on pH as a control parameter, given its biological significance and tight regulation in tissues [19, 20]. By systematically varying pH, we reveal how solution conditions influence the competition between LLCPS and LLPS and identify the regime where ordered nematic tactoids give way to disordered condensates. We note that lysozyme and β‐lactoglobulin were chosen as model systems because they differ in surface properties yet share well‐characterized fibril structure, enabling generalizable insights.

The ability of amyloid fibrils to undergo LLPS as well as LLCPS expands their potential as building blocks for hydrogels and delivery platforms [21]. Whereas anisotropic, ordered phases generated by LLCPS impart directional mechanical stability and organization for scaffolds and optically active materials, isotropic condensates formed through LLPS can yield reversible, soft hydrogels for supramolecular encapsulation. Since pH can drive a transition from LLCPS to LLPS, it can be used as a control mechanism for tuning the balance between structural order and disorder in these materials.

## 2 Results

### 2.1 Characterization of Lysozyme Amyloid Fibrils

**FIGURE 1:**
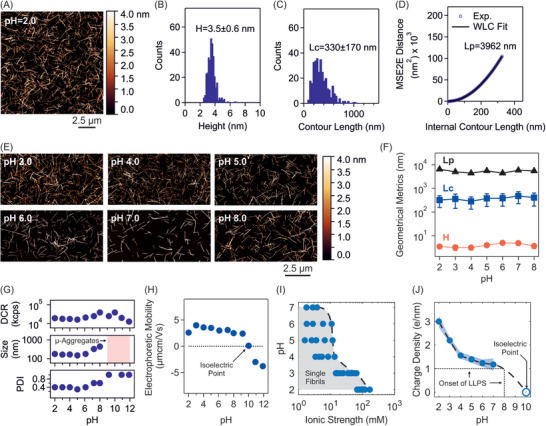
Structural and electrokinetic properties of lysozyme amyloid fibrils (Lys‐A for A to H, Lys‐B for I and J). (A–D) Representative AFM image (A), distribution of height H (B) and contour length Lc (C), and estimation of persistence length Lp (D) at pH 2.0. (E) Representative AFM images across pH range 3.0 to 8.0. (F) Fibril height H, contour length Lc, and persistence length Lp as a function of pH. (G) Derived count rate (DCR), hydrodynamic size and polydispersity index (PDI) as a function of pH. The pH range of “Micro‐Aggregation” is marked. (H) Electrophoretic mobility as a function of pH, showing the isoelectric point of the fibrils at ≈ pH 10. (I) pH versus ionic strength phase diagram in 1:1 electrolyte solution. Shaded area marks the region of stable, single fibril phase. (J) Fibril linear charge density as a function of pH. Shaded area represents 95% confidence interval. The onset of LLPS at pH 8.0 and linear charge density ≈ 1 e/nm are marked with an arrow. Broken line in (I) and (J) is a spline function that serves as a guide to the eye. Error bars and "±" represent standard deviation.

The morphology of lysozyme amyloid fibrils was investigated using AFM imaging. A representative image at pH 2.0, together with analysis of height *H*, contour length *L_c_* and persistence length *L_p_* are shown in Figure 1A–D. At this pH, the fibrils were characterized by an aspect ratio $L_{c}/D∼10^{2}$ and a reduced persistence length $L_{p}/L_{c}∼10^{1}$, indicative of a semiflexible to rigid structure. The pH of fibril solution was slowly adjusted to higher pH values using dialysis. Representative AFM images of fibrils across the pH range 3.0 to 8.0 are shown in Figure 1E. Analysis showed consistent properties (including aspect ratio and reduced persistence length) across pH 2.0 to 8.0 (Figure 1F), indicating structural stability at higher pH values (see also ThT assay in Figure S1‐9 and CD in Figure S1‐10). Specifically, the preserved reduced persistence length reflects the inherently high bending elasticity of the fibril structure [2].

We then investigated the colloidal stability of fibrils as a function of pH using DLS. The sensitivity of DLS to aggregation arises from the sixth‐power dependence of the scattered intensity on particle size. In the pH range 2.0 to 6.0, fibrils exhibited stable scattered intensity, expressed by derived count rate (DCR), and hydrodynamic size and a lower polydispersity index (PDI) (Figure 1G). At pH 7.0 and 8.0, a noticeable increase in DCR and size indicated formation of small aggregates (size < 1 µm), and from pH 9.0 to 12.0, microaggregates (size > 1 µm). We then evaluated electrophoretic mobility of fibrils in the pH range 2.0 to 12.0 and found the isoelectric point to be near pH 10.0 (Figure 1H) [17]. We then constructed the pH versus ionic strength phase diagram of fibrils which indicated stability up to about 200 mM in 1:1 electrolyte solution at pH 2.0, but that the stability window shrank progressively at higher pH values. The fibril linear charge density at each pH was subsequently obtained from electrophoretic mobility measurements using the theoretical framework described in SI 3, which relates mobility to surface potential and charge density through the nonlinear Poisson–Boltzmann and generalized Henry formulations. The linear charge density showed a monotonic decrease by a factor of about 3 from pH 2.0 to pH 7.0 (Figure 1J and Table S1‐1) [22].

### 2.2 Dynamics and Spatial Arrangement of Lysozyme Amyloid Fibrils across LLCPS at pH 2.0

**FIGURE 2:**
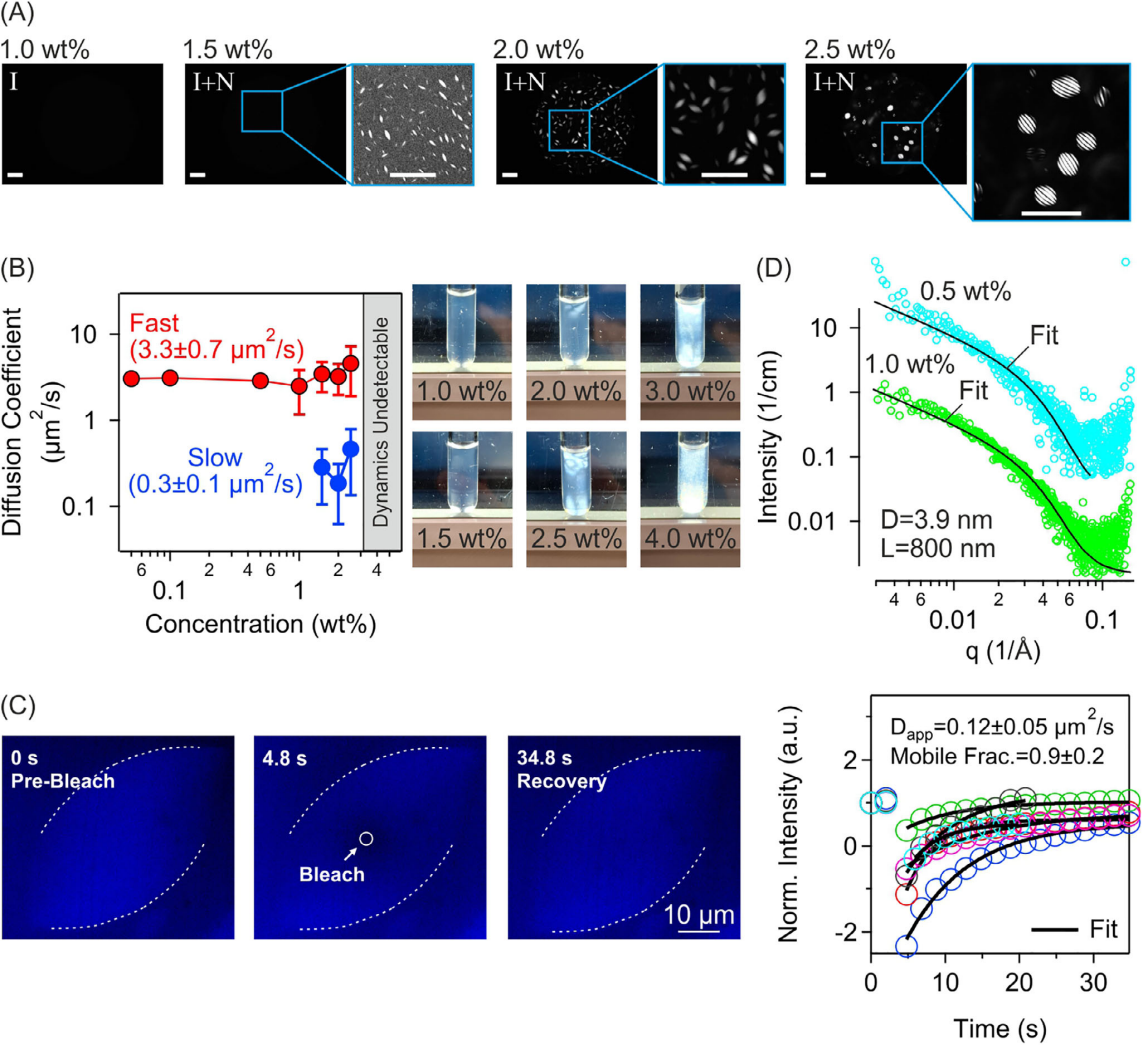
LLCPS in lysozyme amyloid fibrils Lys‐A solutions at pH 2.0. (A) Representative cross‐polarized microscopy images showing isotropic (I) phase at 1.0 wt %, and isotropic‐nematic coexistence (I+N) at 1.5, 2.0, and 2.5 wt %. Magnified regions highlight tactoid structures. Scale bar: 20 µm. (B) Diffusion coefficient of fibrils as a function of concentration measured by 3D cross‐correlation DLS. Error bars represent standard deviation. Inset: Photographs of 3D‐DLS glass tubes containing amyloid fibril solutions, placed between two crossed linear polarizers. (C) A representative bleach‐recovery experiment on a tactoid (border with the isotropic surrounding is partially marked with a broken line for clarity). After bleaching at time 4.8 s (circle), recovery was monitored over a period of 20 to 35 s. Normalized intensity versus time of six independent tactoids is shown together with fits. An average apparent diffusion coefficient ***D***_**app**_ and mobile fraction are reported. (D) SAXS profiles of amyloid fibril solutions at 0.5 and 1.0 wt % together with fits to cylindrical form factor model. Fit values of fibril diameter ***D*** and length ***L*** are indicated.

Transition from isotropic to isotropic‐nematic coexistence in lysozyme amyloid fibril solutions at pH 2.0 was investigated using cross‐polarized microscopy. Representative images in Figure 2A show an isotropic solution at 1.0 wt % (equivalent to 10 mg/ml for an assumed solution density of 1.0 g/ml), and isotropic‐nematic coexistence at 1.5, 2.0, and 2.5 wt %, whereby LLCPS occurred between 1.0 and 1.5 wt %. At 1.5 wt %, small, homogeneous tactoids were present but they were less discernable in the images. At 2.0 wt %, larger, more distinct homogeneous and bipolar tactoids, and at 2.5 wt %, cholesteric tactoids were found [23]. The internal structure of tactoids is known to evolve with both the volume and the concentration of the tactoids and LLCPS encompasses a vast range of tactoid symmetries [24, 25].

The dynamics of fibrils in isotropic and isotropic‐nematic coexistence was then investigated using 3D cross‐correlation DLS (Figure 2B). From 0.05 to 1.0 wt %, a single diffusion constant, corresponding to free diffusion of fibrils, was measured. At 1.5 wt % and above, two distinct dynamic modes appeared; a fast mode with the same diffusion constant as measured at the lower concentrations, and a slower mode with an order‐of‐magnitude lower diffusion constant, likely associated with restricted fibril dynamics within the tactoids. This observation was further corroborated by FRAP bleach‐recovery experiments on tactoids. The apparent diffusion coefficient of fibrils inside tactoids was in the same range as the slow diffusion coefficient measured by DLS (Figure 2C and Video S1). Specifically, the emergence of the slow dynamic mode coincided with the bifurcation concentration identified by crossed‐polarized microscopy (Figure 2A). Above 3.0 wt %, the intensity cross‐correlation function was no longer measurable, potentially because the isotropic phase had shrunk significantly or the system had transitioned to a fully nematic phase. At these concentrations, cross‐polarized microscopy showed the emergence of complex crystalline phases (Figure S1‐2). These observations were further corroborated by placing the 3D‐DLS glass tubes between two crossed linear polarizers, where birefringence was visible at 2.0 wt % and above (Figure 2B).

The spatial arrangement of fibrils was then investigated using SAXS (Figure 2D). In isotropic solutions at 0.5 and 1.0 wt%, the measured intensity as a function of momentum transfer *q* was consistent with rigid cylinder form factor. Fitting the profiles yielded fibril dimensions in agreement with AFM results (Figure 1B,C).

**FIGURE 3:**
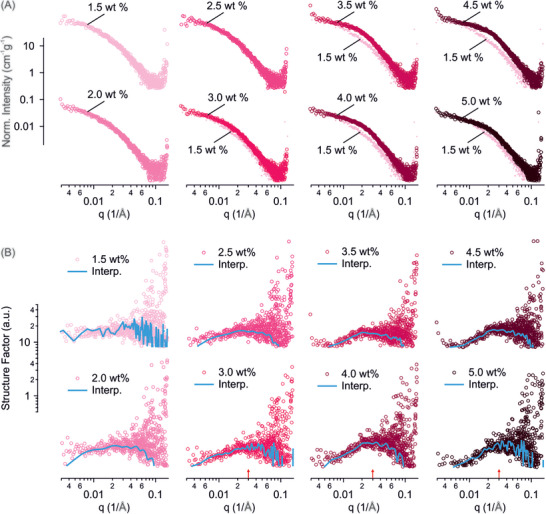
Inter‐fibril correlations in lysozyme amyloid fibrils Lys‐A solutions at pH 2.0 and above the isotropic concentration. (A) SAXS intensity profiles normalized (Norm.) to respective concentrations in the range 1.5 to 5.0 wt %. The normalized intensity profile of 1.5 wt % is replicated under the normalized intensity profiles of 3.0 to 5.0 wt %. (B) Profiles of structure factor in the same concentration range. Blue lines show interpolations (Interp.) Peak location at $q≅0.03\;{Å}^{-1}$ is indicated by red arrow on x‐axis.

SAXS profiles at concentrations 1.5 to 5.0 wt % were collected and normalized to their respective concentrations as shown in Figure 3A. At 1.5 to 2.5 wt %, the normalized scattering profiles did not display detectable long‐range order; however, as the concentration increased from 3.0 to 5.0 wt %, the profiles exhibited a pronounced upturn at intermediate *q* range, 0.01 to 0.04 Å^−1^, which became evident when compared with profile of the solution at 1.5 wt %, suggesting detectable inter‐fibril correlations and ordering associated with nematic and chiral nematic (cholesteric) domains. The structure factor was calculated from *S* (*q*) = *I*(*q*)/(*N* × *P*(*q*)) , where *I*(*q*) is the scattering intensity, *N* the fibril density, and *P*(*q*) the form factor (Figure 3B). The form factor *P*(*q*) was estimated from the scattering of the isotropic solution at 1.0 wt % (Figure 2D). At 1.5 to 2.0 wt %, *S*(*q*) remained close to unity. However, with increasing concentration from 3.0 to 5.0 wt%, *S*(*q*) developed a broad peak in the same intermediate *q* range mentioned above. Interpolation indicated that the peak centered at $q≅0.03\;{Å}^{-1}$, corresponding to a spatial periodicity (inter‐fibril spacing) of about 20.9 nm. In comparison, solutions of long, semi‐flexible β‐lactoglobulin amyloid fibrils were reported to have a structure factor peak at $q≅0.05\;{Å}^{-1}$ at 1.7 wt % and $q≅0.06\;{Å}^{-1}$ at 13.7 wt %, corresponding to spatial periodicities of about 12.6 nm and 10.5 nm, respectively [26]. We note that the larger inter‐fibril spacing observed with shorter, more rigid lysozyme fibrils is attributed to a higher charge density (3.0 e/nm for lysozyme and 2.3 e/nm for β‐lactoglobulin amyloid fibrils, Tables S1‐1 and S2‐1) and a lower configurational entropy ($L_{p}/L_{c}∼10^{1}$ for lysozyme and ≈ 10^−1^ for long β‐lactoglobulin amyloid fibrils [2]).

### 2.3 Transition from LLCPS to LLPS in Lysozyme Amyloid Fibril Solutions

**FIGURE 4:**
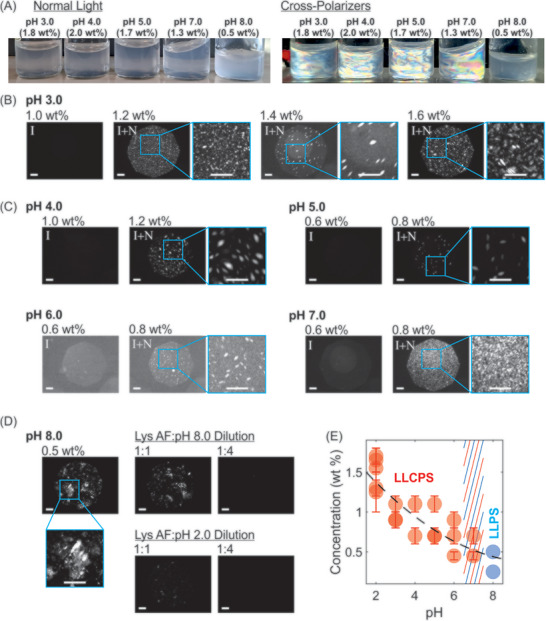
LLCPS and LLPS in lysozyme amyloid fibrils Lys‐A solutions. (A) Photographs of solutions at different pH values under normal light (left) and between two crossed linear polarizers (right). Glass tube diameter = 1 cm. (B) Representative cross‐polarized microscopy images at pH 3.0, isotropic (I) at 1.0 wt %, and isotropic‐nematic coexistence (I+N) at 1.2, 1.4, and 1.6 wt %. Scale bar: 20 µm. (C) Representative cross‐polarized microscopy images at pH 4.0, 5.0, 6.0, and 7.0 at concentrations below LLCPS (I) and above LLCPS (I+N). Scale bar: 20 µm. (D) Representative cross‐polarized microscopy images at pH 8.0 showing large, and birefringently irregular condensates at 0.5 wt % (Left). Additional images (Right) show reversibility to isotropic phase of LLPS upon dilution at pH 8.0 (ratios 1:1 and 1:4), and upon acidification by pH 2.0 (ratios 1:1 and 1:4). Lys AF: lysozyme amyloid fibril. Scale bar: 20 µm. (E) Phase diagram of concentration versus pH at the start of LLCPS and onset of LLPS, as determined by cross‐polarized microscopy. Hatched area at pH 7.0 marks extrapolated transition from LLCPS to LLPS. Error bars represent observation window between I and I‐N coexistence concentrations. Dashed line is a guide to the eye through mean values (see main text).

Transition from LLCPS at pH 2.0 to LLPS at higher pH values was subsequently investigated. Representative photographs of samples in glass tubes under normal illumination and between two crossed linear polarizers at pH 3.0 to 8.0 are shown in Figure 4A. From pH 3.0 to 8.0, the solutions gradually changed from transparent to translucent under normal light. At pH 3.0 to 7.0, the solutions were birefringent between two crossed linear polarizers, indicative of liquid crystalline domains in these solutions. At pH 8.0, the structural colors were not evident. To gain insight into the evolution of phase behavior with pH and concentration, the samples were investigated using cross‐polarized microscopy. Representative examples are shown in Figure 4B–D. At pH 3.0, a transition from isotropic to isotropic‐nematic coexistence was observed between 1.0 and 1.2 wt %, where the coexistence was characterized by tactoids visible at 1.2, 1.4 and 1.6 wt % (Figure 4B). Similarly, at pH 4.0 and 5.0, the solutions displayed LLCPS, respectively between 1.0–1.2 wt % and 0.6–0.8 wt % (Figure 4C). At pH 6.0, the tactoids formed between 0.6–0.8 wt % but were less evident (Figure 4C). At pH 7.0, individual tactoids were no longer resolved; the sample was nevertheless birefringent, which may be due to the presence of numerous small, possibly homogeneous tactoids (Figure 4C).

At pH 8.0, crossed‐polarized microscopy revealed the formation of large, irregular microscopic condensates, which exhibited multiple birefringent domains, but the condensates lacked a well‐defined nematic order (a continuous director field) observed with tactoids at the lower pH values of 2.0 to 6.0 (Figure 4D). These objects appear to have formed through LLPS, although we were not able to observe the early nucleation stage of the process. Dilution experiments while maintaining pH 8.0 revealed reversibility of the condensates to isotropic phase (Figure 4D). Similarly, returning the solution to acidic condition by diluting with pH 2.0 led to transition to isotropic phase, although at a lower dilution factor compared to dilution at pH 8.0.

Figure 4E gives a summary of multiple measurements along the lines of those observations presented in Figure 4B–D. From these measurements, LLCPS was observed to occur at the mean (minimum) concentrations of 1.49 (1.25) wt %, 0.97 (0.90) wt %, 0.90 (0.70) wt %, 0.83 (0.70) wt %, 0.68 (0.45) wt %, and 0.58 (0.45) wt %, respectively at pH 2.0, 3.0, 4.0, 5.0, 6.0, and 7.0. We find that the critical concentration for LLCPS decreased with pH, until near the isoelectric point, where the system exhibited LLPS (Figure 4E). At the onset of LLPS, the linear charge density of fibrils was estimated to be around 1 e/nm (Figure 1J).

**FIGURE 5:**
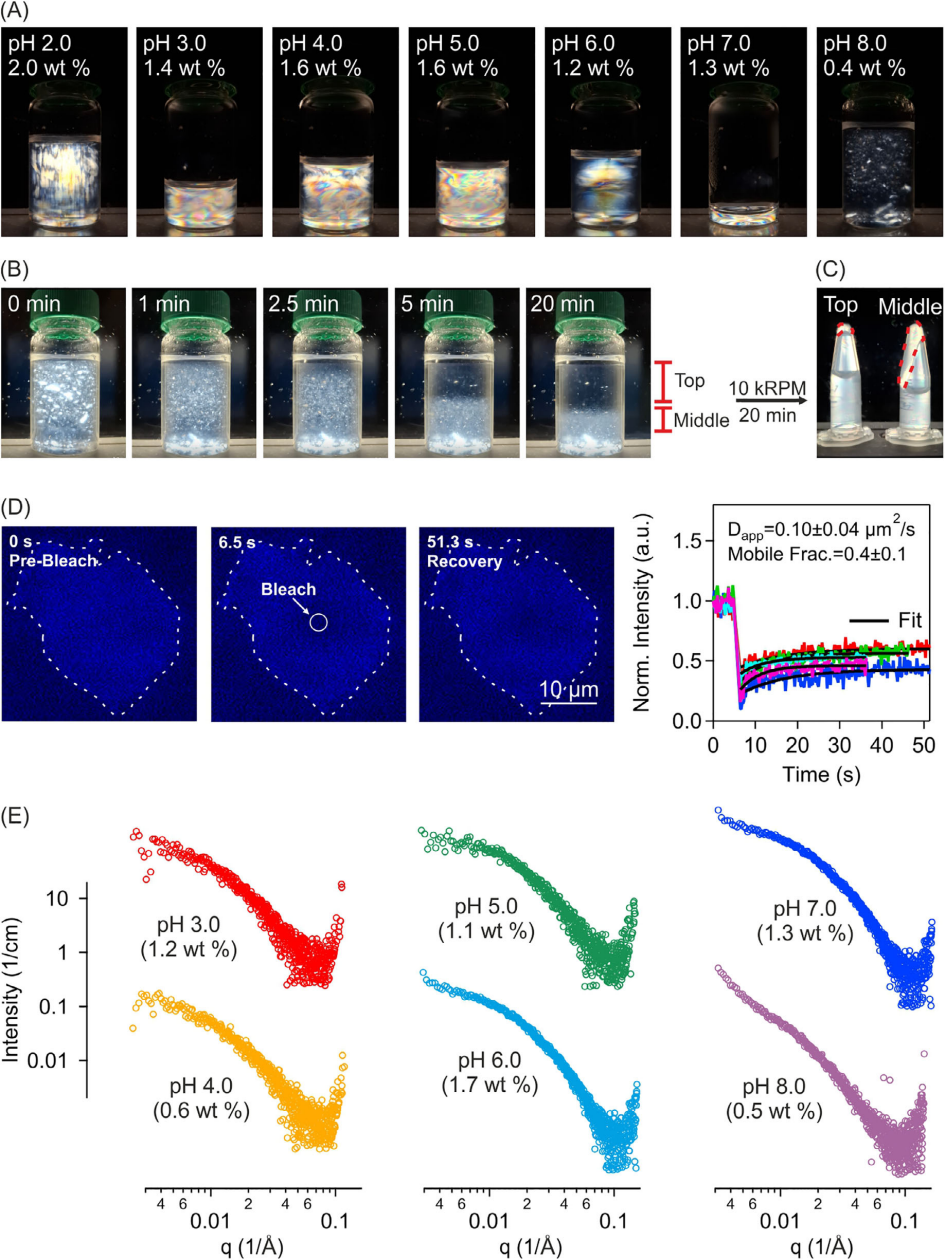
Phase behavior and SAXS profiles of lysozyme amyloid fibrils at varying pH. (A) Photographs under crossed linear polarizers showing structural colors for Lys‐B samples at pH 2.0–7.0, and the appearance of dispersed birefringent condensates at pH 8.0. Concentrations (wt %) are indicated for each condition. (B) Time series of Lys‐B sample at pH 8.0, settled for 0–20 min, resulting in a dilute top fraction (marked “Top”) and two denser middle (marked “Middle”) and bottom fractions. (C) Aliquots collected from Top and Middle were subjected to centrifugation and imaged under crossed linear polarizers. The condensates coalesced (marked with broken red lines). (D) A representative bleach‐recovery experiment on a condensate of Lys‐B at pH 8.0. After bleaching at time 6.5 s (circle), recovery was monitored over a period of 30 to 50 s. Normalized intensity versus time of five independent condensates is shown together with fits. An average apparent diffusion coefficient ***D***_**app**_ and mobile fraction are reported. (E) SAXS profiles of Lys‐A solutions at pH 3.0 to 8.0.

The birefringent characteristics across pH 2.0–7.0 and the LLPS behavior at pH 8.0 were consistent in different sample preparations. Under crossed linear polarizers, structural colors were observed from pH 2.0 to 7.0, whereas at pH 8.0 distinct birefringent condensates appeared (Figure 5A). The sample at pH 8.0 was allowed to settle for 20 min, producing a dilute top fraction and two denser middle and bottom fractions. Aliquots from the top and middle fractions were centrifuged and re‐imaged under linear crossed polarizers. The measurements revealed that the condensates had coalesced into larger birefringent domains (Figure 5B,C). This behavior was consistent with a fluid‐like property of the condensates. This interpretation was further corroborated by FRAP bleach‐recovery measurements on the condensates (Figure 5D and Video S2). Fluorescence recovery showed a comparable apparent diffusion coefficient to recovery in tactoids at pH 2.0 (c.f. Figure 2C), indicating fluid‐like behavior. The more pronounced difference lied in the mobile fraction, which was approximately twice as large at pH 2.0 compared to pH 8.0. This reduced mobile fraction is compatible with fibrils bound in an attractive interaction potential at pH 8.0, imparting an apparent elasticity to the condensates.

Fibril structure across the same pH range was then investigated using SAXS (Figure 5E), and distinct differences were observed between the scattering profiles at pH 3.0 to 7.0 and the profile at pH 8.0. In particular, while the intensity curves at pH 3.0 to 7.0 exhibited the characteristic shape observed at pH 2.0 (Figures 2 and 3), at pH 8.0, the intensity profile displayed a pronounced upturn at low q values < 0.02 Å^−1^. This upturn is indicative of fibril organization in condensates and coincides with the onset of LLPS at pH 8.0 (Figure 4D).

### 2.4 Phase Behavior, Structure and Dynamics of β‐Lactoglobulin Amyloid Fibrils

β‐lactoglobulin amyloid fibrils have been extensively investigated by our group [24, 26, 27, 28, 29]. Here, we summarize their structure, dynamics, and phase behavior during transition from LLCPS to LLPS, and we leave to the Supporting Information SI 2 a more extended discussion. The fibrils were rigid (L_p_/L_c_ ≫ 1), with an approximately consistent height, contour length, and persistence length across pH 2.0–4.0 (Figure S2‐2). Similarly, the hydrodynamic size of the fibrils remained unchanged within this pH range; however, at pH 5.0 and 6.0, microaggregates formed (Figure S2‐3). The transition from isotropic to isotropic‐nematic coexistence at pH 2.0 had the typical concentration dependence reported previously [24, 26, 27, 28, 29] and was corroborated, in addition to cross‐polarized microscopy, by the emergence of slow fibril dynamics in the coexistence region, as well as the absence of structure factor in the isotropic solutions (Figures S2‐4–S2‐6, D2‐8 and S2‐9). At pH 3.0 and 4.0, birefringent domains were apparent between two crossed linear polarizers (Figure S2‐10). Additionally, cross‐polarized microscopy combined with the analysis of standard deviation in grayscale intensity confirmed LLCPS, although the transition was more prominent at pH 3.0 than pH 4.0 (Figure S2‐11). At pH 5.0, the fibril solution transitioned to LLPS. In particular, at this pH, bulk birefringence between two crossed linear polarizers disappeared, while birefringent condensates became visible (Figure S2‐10). Subsequently, under cross‐polarized microscopy, the condensates were found to contain multiple birefringent domains, lacking a coherent nematic order (Figure S2‐13). These LLPS structures were partly reversible to the isotropic phase by dilution in pH 5.0 and were fully reversible to the isotropic phase by acidification (Figure S2‐12). Similar to lysozyme amyloid fibrils (Figure 4E), the critical LLCPS concentration was found to decrease with increasing pH, until the onset of LLPS at pH 5.0 (Figure S2‐14). SAXS profiles were superimposable at pH 2.0 and 3.0; however, deviations appeared in the profiles at pH 4.0 and 5.0, suggesting changes in inter‐fibril arrangements (Figure S2‐15). This observation was consistent with a gradual crossover from LLCPS to LLPS with increasing pH.

## 3 Discussion

### 3.1 Summary of Key Findings

**FIGURE 6:**
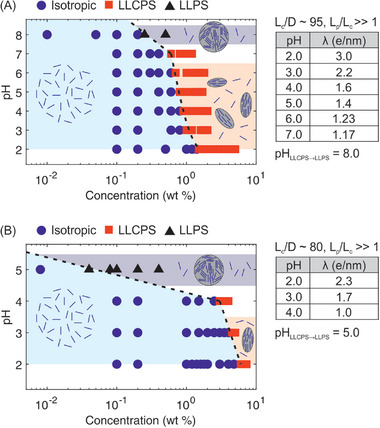
Phase diagrams of (A) lysozyme amyloid fibrils Lys‐A (aspect ratio ≈ 95) and (B) β‐lactoglobulin amyloid fibrils Blg‐C (aspect ratio ≈ 80) as a function of pH and fibril concentration (wt %). The diagrams illustrate transitions from isotropic dispersion (blue circles in the light blue shaded region) to LLCPS (red squares in the light red shaded region) and LLPS (black triangles in the light purple shaded region). A transition region between LLCPS and LLPS is extrapolated between the two regimes (pH 7.0 for lysozyme and pH 4.0 for β‐lactoglobulin fibrils, not shaded). Schematic insets depict representative microstructures for each region: dispersed fibrils (isotropic), biphasic tactoid structures (LLCPS), and birefringent microscopic condensates lacking a coherent nematic order (LLPS). Tables to the right give a summary of important physical properties of each fibril.

The phase behavior of amyloid fibril solutions composed of lysozyme or β‐lactoglobulin was investigated with a focus on interplay between LLCPS and LLPS. Lysozyme fibrils were rigid, with an aspect ratio of about 95 (Lys‐A), and linear charge density varying from 3.0 to 1.17 e/nm at pH 2.0 to 7.0. β‐Lactoglobulin fibrils were also rigid, with an aspect ratio of about 80 (Blg‐C), and linear charge density varying from 2.3 to 1.0 e/nm at pH 2.0 to 4.0. LLCPS and LLPS took place in solutions of pre‐assembled amyloid fibrils whose molecular structure remained stable despite pH variation (see for lysozyme amyloid fibrils: Figure 1F; Figure S1‐9 and 1‐10). The phase diagrams presented in Figure 6 provide a comprehensive overview of pH‐ and concentration‐dependent phase behavior of fibrils. The phase diagrams illustrate the existence of three regions: isotropic region at low concentration and low pH, isotropic‐nematic coexistence region at high concentration and low pH, and LLPS region at high concentration and high pH. The fibril aspect ratio and the charge density set the position and length of the phase boundaries.

Two principal findings emerged: (1) a progressive decrease in the start concentration for LLCPS with increasing pH, and (2) a transition from LLCPS to LLPS near a critical threshold pH (pH 8.0 for lysozyme and pH 5.0 for β‐lactoglobulin fibrils). Findings (1) and (2) became evident in our investigations through a slow pH elevation process by dialysis. These findings show how common solution conditions can tune the phase state and microstructure of fibril condensates, revealing an interplay between liquid crystalline ordering (LLCPS) and isotropic phase separation (LLPS).

### 3.2 Mechanistic Insights

The observed phase transitions can be initially attributed to pH‐dependent changes in fibril charge density: increasing pH reduces the net charge density and diminishes electrostatic repulsion. This facilitates inter‐fibril approach, shifting the free energy of interactions from favoring LLCPS (driven by entropy) to LLPS (driven by enthalpy). However, across a wider pH range, LLCPS dominated and tactoids were formed, and when LLPS became apparent, phase‐separated structures contained multiple birefringent domains without a coherent nematic order. Since the condition *L_p_*/*L_c_* ≫ 1 persisted across all pH conditions, variations in fibril rigidity played no significant role in the transition mechanism.

### 3.3 LLCPS

An isotropic solution of thin, rigid, charged rods transitions to isotropic‐nematic coexistence above a threshold volume fraction ϕ_I‐N_ ≡ (1‐0.75*h*)^−1^(*D* ^2^/*LD*_eff_), driven by excluded volume interactions [30, 31]. The effective diameter D_eff_ exceeds the physical fibril diameter D by κ^−1^(lnA′ +C_E_+ ln2‐0.5), where κ^−1^ is the Debye length, A′ the electrostatic interaction amplitude, and C_E_ =  0.577 (Euler's constant) [30, 31]. The twisting factor h  =(κD_eff_)^−1^  penalizes parallel alignment (nematic order) [32]. For weakly charged rods (λQ <1), where λ is the linear charge density and Q the Bjerrum length (0.7 nm in water at 23°C), Debye‐Hückel approximation gives $A^{\prime }=8\pi \lambda^{2}Qe^{-\kappa D}/\kappa^{3}D^{2}K_{1}^{2}\left(\kappa D/2\right)$, with K_1_ the modified Bessel function of the second kind [32]. From theory increasing D_eff_ lowers the transition concentration, while a higher twisting factor has the opposite effect. Under our experimental conditions, the prefactor in the expression of threshold volume fraction evaluates to 3.659 [10, 30, 31]. To comply with experiments, the volume fraction was converted to weight concentration using c_I − N_ (wt %) = ϕ_I − N_ρ_AF_/[ϕ_I − N_ρ_AF_ + (1 − ϕ_I − N_)ρ_w_]  where ρ_AF_ =  1.3 g/ml and ρ_w_ =  1.0 g/ml are the fibril and water densities (17).

Fibril solutions were prepared at pH 2.0 and dialyzed against water adjusted to a target pH. This approach fixes pH but not the ionic strength, as highly charged fibrils retain counterions through Donnan partitioning [33]. Ion retention causes the internal ionic strength I_in_ to deviate from the nominal ionic strength $I_{\mathrm{nom}}=\frac{1}{2}\;\sum z_{i}^{2}c_{i}$ for H^+^, OH^−^ and their conjugate ions (c.f. Table S1‐3). To account for this effect, I_in_ was iteratively adjusted until the theoretical *c*_I − N_ matched the experimental mean start concentration of LLCPS at each pH (Table S4‐2).

**FIGURE 7:**
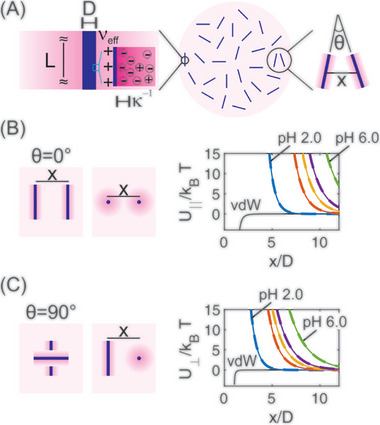
(A) Schematic model of charged rods with diameter ***D*** and length ***L*** in a 1:1 electrolyte solution characterized by Debye length ***κ*** ^−1^. The right panel shows two rods separated by center‐to‐center distance ***x*** and relative orientation angle ***θ***. (B, C) Pair‐interaction potential between two lysozyme amyloid fibrils aligned in parallel (***θ *** =  0°, B) or perpendicularly (***θ *** =  90°, C) as a function of normalized distance ***x***/***D***, for pH 2.0, 3.0, 4.0, 5.0, and 6.0. Only pH 2.0 and pH 6.0 are marked on the figures. The pair‐interaction potential sequentially increases from pH 2.0 to pH 6.0. Solid colored line: electrostatic interaction; black solid line: van der Waals (vdW) interaction; dashed colored line: total interaction potential.

We then used λ and I_in_ at each pH to calculate the pair‐interaction potential between lysozyme fibrils from the sum of electrostatic and van der Waals contributions [34, 35] (Figure 7; SI 5). To calculate the van der Waals contribution, a Hamaker constant of 3kT was assumed [36]. At pH 2.0, the interaction potential is higher in parallel orientation than in perpendicular orientation, giving a relative probability $P=e^{-(U_{∥}-U_{⊥})/\mathrm{kT}}=0.012$ at x_nematic_/D≅5.4, where x_nematic_ corresponds to inter‐fibril spacing in the nematic phase measured by SAXS (Figure 3). At pH 3.0, a lower charge density is offset by an increased Debye length, increasing the electrostatic potential for both orientations. This enlarges the excluded volume (or *D*_eff_, c.f. Table S4‐2), thereby lowering the start concentration for LLCPS, as observed experimentally. Using *P* =  0.012 as a benchmark for nematic ordering, we estimate *x*_nematic_/*D*≅8.5 at pH 3.0. At higher pH values of 4.0, 5.0 and 6.0, the electrostatic interaction potential further increases because the effect of reduced ionic strength overweighs that of reduced linear charge density, shifting the start concentration of LLCPSs to lower values, with estimated inter‐fibril spacings of *x*_nematic_/*D*≅ 9.6, 10.4 and 12.5, respectively. This putative increase in inter‐fibril spacing agrees with the lower fibril concentration required for LLCPS with increase in pH. The parameters used in the calculations of the pair‐interaction potentials are tabulated in Table S4‐2, see also SI 5.

We anticipate that there will be a limit on the minimum I_in_ attainable by dialysis (see Table S4‐2) [33]. This residual ionic strength, in combination with a linear charge density below about 1 e/nm at pH 8.0 (Figure 1J), shifts the phase behavior of lysozyme amyloid fibrils towards LLPS, where attractive interactions dominate the phase behavior.

### 3.4 LLCPS Versus LLPS in Solutions of Amyloid Fibrils

The theoretical model presented above assumes homogeneous, rigid rods with uniformly distributed surface charge. In practice, heterogeneous or patchy interactions, such as locally hydrophobic stretches, asymmetric charge distributions, or surface roughness arising from flanking unstructured domains, can generate additional attractive forces that may shift critical LLCPS boundaries or LLCPS/LLPS transition. In addition, nonfibrillar protein fragments or partially aggregated oligomeric domains can modulate the effective interaction potential and alter the observed phase thresholds. The gradual transition of the start concentration of LLCPS, and transition to LLPS suggest that such minor species had minimal impact on the observed behaviors and these transitions are governed by surface properties of lysozyme and β‐lactoglobulin fibrils.

The distinction between LLCPS and LLPS lies in their dominant driving forces and the nature of the resulting phases. LLPS typically occurs in solutions of flexible macromolecules, where phase separation is governed by the balance between the entropy of mixing and the enthalpy of interaction, characterized by the Flory parameter χ [37]. When χ exceeds a critical value, attractive interactions overcome mixing entropy, leading to the formation of isotropic liquid droplets [10, 11]. LLCPS, which is the canonical phase transition model for rigid charged rods, results from the competition between orientational entropy loss and excluded‐volume gain upon alignment [30]. LLCPS produces anisotropic, birefringent tactoids [9, 30].

Our results indicate that, while LLCPS remains the dominating mechanism in amyloid fibril suspensions, LLPS can emerge under specific environmental conditions. As the pH was increased from a regime favoring LLCPS toward the isoelectric point, we observed the formation of microscopic condensates containing multiple randomly oriented birefringent domains, lacking a coherent nematic order. The onset pH of LLPS depends on fibril charge density and solution ionic strength and was found to be pH 8.0 for lysozyme amyloid fibrils and pH 5.0 for β‐lactoglobulin amyloid fibrils. These observations suggest that parallel alignment is locally maintained during LLPS, but rather that this is driven by a directional attractive interaction potential among fibrils, rather than excluded volume. Distinct birefringent assemblies then conjugate to form large, disordered condensates which were reversible to isotropic phase upon dilution or reacidification. The transition from LLCPS to LLPS appeared to be first‐order, as revealed by the coexistence region of two phases, one dilute and isotropic and the other dense and in the form of polycrystalline, birefringent condensates.

We note a distinction between multigrain liquid crystalline domains resulting from LLCPS and the microscopic condensates reported here for lysozyme amyloid fibrils at pH 8.0 and β‐lactoglobulin amyloid fibrils at pH 4.0. In systems undergoing LLCPS, multigrain liquid crystalline domains can appear at the late stages resulting from tactoid coalescence [24]; however, these structures are preceded by a well‐defined sequence of tactoidal morphologies including homogeneous, bipolar, and, in the case of chiral mesogens such as amyloid fibrils and nanocellulose, cholesteric tactoids. These multigrain liquid crystalline domains are further characterized by focal conic defects and interference bands [24]. In contrast, the LLPS condensates reported here showed no intermediate tactoidal states or birefringent interference features but instead displayed coalesced birefringent domains without a coherent nematic order. The absence of any identifiable tactoids supports our classification of this regime as LLPS rather than LLCPS.

The non‐pathogenic model proteins employed in this work allowed reproducible amyloid fibrils formation and controlled investigations of their phase behavior. The revealed physical principles, including variable start concentration of LLCPS with pH and the competition between LLCPS and LLPS, are expected to extend to pathological amyloids such as Aβ and Tau, where mature fibrils (*L*/*D* ≫ 1) at comparable fibrillization stages (*L*_p_/*L* ≫ 1) reach concentrations above the isotropic–nematic coexistence threshold or are trapped in an attractive interaction potential. We note however that investigation of LLPS and LLCPS transitions in mature Aβ and Tau fibrils has not yet been documented.

## 4 Conclusions

Our observations indicate that the phase behavior of amyloid fibrils is a result of a complex interplay between LLCPS and LLPS depending on simple environmental conditions. We observed dominance of LLCPS over LLPS over a wide pH range, but the ordering (LLCPS) and coarsening (LLPS) transitions appeared to be separable and tunable. Entropy drives LLCPS, and when environmental parameters shift the balance between entropic and attractive enthalpic contributions, they lead to LLPS. Although our study focused on artificial model fibrils, the results may be relevant to functional and pathological amyloid fibrils, in which phase separations contribute to structural and functional properties. This study provides mechanistic insight into phase competition in amyloid fibril systems under simplified in vitro conditions. Biological environments contain additional factors, including crowding agents, and macromolecular interactions, that may influence phase behavior in a more complex manner. Future studies should explore these effects with particular attention to LLCPS and LLPS interplay.

## 5 Experimental Section

### 5.1 Preparation of Lysozyme and β‐Lactoglobulin Amyloid Fibrils

Lysozyme from chicken egg white (crystalline powder, 70000–140000 U/mg, Product 62971, Sigma‐Aldrich), and β‐lactoglobulin purified from whey protein isolate (NZMP, New Zealand) [38], were used to prepare amyloid fibrils. Protein powder was dissolved in Milli‐Q water adjusted to pH 2.0 at a concentration of 2.0 wt %. The dispersion was filtered through a 0.22 µm polyethersulfone filter (Millipore) and heated at 90°C under constant stirring for 24 h for lysozyme and 5 h for β‐lactoglobulin, after which the fibrillization was quenched by immersion in an ice‐water bath. The resulting amyloid fibril solution was centrifuged at 10,000 RCF for 20 min to remove potential gels. To obtain rigid amyloid fibrils, the solution was mechanically sheared using a stainless‐steel stick blender (emerio.eu) at speed setting I or II (AFM imaging did not show a significant fibril length dependency on the speed setting). The shearing procedure of 500 µl amyloid fibril solution (2.0 wt %) in 3000 ml glass beaker (diameter 13.5 cm, height 28 cm, SIMAX) consisted of two 45‐s blending steps, separated by about 15‐min resting period to allow the foams to dissipate. After blending, the solution was left overnight to settle at room temperature. The solution was then dialyzed at 4°C for 5 days against Milli‐Q adjusted to pH 2.0 with daily changes of the dialysis bath. For dialysis, 100 kDa MWCO Spectra/Por dialysis membranes (Repligen) were used. The solution was then concentrated by reverse osmosis at 4°C for several days against 10 wt % polyethylene glycol (BioUltra, Mr ≈ 35,000) solution at pH 2.0. Here, 6–8 kDa MWCO Spectra/Por 1 dialysis membranes (Repligen) were used. pH adjustment to 2.0 were by using hydrochloric acid (HCl, 37%, VWR). Two independent lysozyme amyloid fibril samples, labelled Lys‐A and Lys‐B, were prepared. Four independent β‐lactoglobulin amyloid fibril samples, Blg‐A, Blg‐B, Blg‐C and Blg‐D, were prepared. Specific investigations with these samples were reported in SI 1 and 2.

### 5.2 pH Adjustment for Phase Transition Measurements

pH of amyloid fibril solutions from the initial value of pH 2.0 to pH 3.0 to 8.0 for lysozyme and to pH 3.0 to 5.0 for β‐lactoglobulin were adjusted by dialysis against Milli‐Q water adjusted to the desired pH. For dialysis, Float‐A‐Lyzer 3.5‐5KD (Repligen) dialysis devices were employed. The dialysis bath was changed daily, and pH of amyloid fibril solution inside the dialysis device was monitored using a micro pH meter (Hamilton). pH adjustments were by using hydrochloric acid (HCl, 37%, VWR) and sodium hydroxide (NaOH, Sigma‐Aldrich).

### 5.3 Atomic Force Microscopy (AFM) Measurements

AFM was used to characterize the morphology of amyloid fibrils. A 10 µl aliquot of amyloid fibril solution (≤ 0.01 wt %) was deposited onto freshly cleaved mica (10 mm diameter, Ted Pella). The sample was incubated for 2 min, then gently rinsed with 1 ml of Milli‐Q water at the same pH as the amyloid fibril solution and dried under a gentle stream of air. AFM imaging was carried out using MultiMode 8 Scanning Probe Microscope (Bruker) in soft tapping mode under ambient conditions, and with RTESPA‐150 cantilevers (Bruker). The oscillation amplitude was set to ≈90% of the free oscillation amplitude at a frequency near the natural resonance frequency of the cantilever. Images were flattened using NanoScope Analysis 3.0 (Bruker) or Gwyddion 2.47, and further analyzed for statistical characterization using FibrilApp [39]. The persistence length Lp was obtained from fit to worm‐like chain (WLC) model of the mean squared end‐to‐end distance of the fibrils [39].

### 5.4 Cross‐Polarized Optical Microscopy Measurements

Cross‐polarized optical microscopy was used to investigate the phase behavior of amyloid fibril solutions. Samples were prepared using two types of spacer frames. Square adhesive frames, 250 µm thick (Gene Frame, Thermo Scientific), and circular adhesive frames, 120 µm thick (Grace Bio‐Labs SecureSeal, Merck) were used. Standard glass microscope sides (Brand, Germany) were cleaned with detergent and hot water, rinsed thoroughly with deionized water and Milli‐Q water, and then dried with an airstream. Spacer frames were adhered to the slides, and the amyloid fibril solutions were introduced into the wells. Coverslips were placed over the wells to hermetically seal the samples. Microscopy was performed using a Zeiss Axio Imager M1m microscope equipped with an AxioCam MRc camera. Köhler illumination was adjusted, and the rotation of the polarizer and analyzer was calibrated prior to imaging to ensure optimal polarization. The aperture diaphragm was occasionally reduced to enhance image contrast. The microscope stage allowed 360° rotation, enabling imaging at various sample orientations. A 5× objective lens with a numerical aperture of 0.12 was used. Images were analyzed using ImageJ [40].

For bulk observation of transparency or birefringence at each pH value, glass tubes with a diameter of 1 cm were used, respectively under normal illumination or between two crossed linear polarizers.

### 5.5 Electrophoretic Mobility and Dynamic Light Scattering (DLS) Measurements

(A) Analysis of isoelectric point: pH of amyloid fibril solutions (0.1 wt %) was adjusted in the range 2.0–12.0 by incremental addition of NaOH. (B) Analysis of pH–ionic strength phase diagram and computation of linear charge density: pH of amyloid fibril solutions was adjusted in the pH range below the isoelectric point by dialysis and then NaCl was added to obtain a desired ionic strength increment, from 1 to 200 (mM). The final fibril concentration for the measurements was 0.25 wt %. See SI 3 for theoretical treatment of this data. Samples were loaded into disposable folded capillary electrophoresis cells (DTS1070, Malvern Panalytical) and transferred to Malvern Zetasizer Nano ZS (Malvern Panalytical). Electrophoretic mobility measurements were performed with an applied voltage set manually to 40 V. Each sample was measured five times (A) or twelve times (B) with a 1‐min equilibration period before each measurement. To verify that the electric field applied during electrophoresis did not alter the amyloid fibrils, DLS measurements of size and polydispersity index were performed in the same cell before and after the electrophoresis measurements using the same device. DLS was conducted at a fixed angle of 173°, with each measurement consisting of three runs of up to 30 seconds. The time correlation function of scattered intensity was analyzed using CUMULANT method by Zetasizer software (Malvern). Water properties were used as the dispersant for all measurements. All measurements were performed at 23°C. Average and standard deviation are reported.

### 5.6 3D Cross‐Correlation Dynamic Light Scattering (3D Cross‐Correlation DLS) Measurements

Dynamics of amyloid fibrils at various concentrations at pH 2.0 were investigated using 3D cross‐correlation DLS (LS Instruments). The instrument was equipped with a He−Ne laser emitting polarized light at a wavelength of 632.8 nm. The laser beam was split, and the cross‐correlation of the scattered intensity from the two beams, probing the same sample volume, was calculated. Samples were loaded in 1 cm diameter glass capillaries and observed between two crossed linear polarizers before the light scattering measurements. Measurements were conducted at scattering angles ranging from 40° to 140° in 10° increments, with each angle measured over a 20‐min period. The time correlation function of the scattered intensity was analyzed using CONTIN method [41], which enabled distinction between fast and slow diffusion processes, the latter arising from nematic ordering of the fibrils [26]. Average and standard deviation of diffusion constants over the measured scattering angle range are reported.

### 5.7 Fluorescence Recovery after Photobleaching (FRAP)

Thioflavin T (ThT, Sigma‐Aldrich) from a stock solution (20 mM in DMSO) was diluted to 4 mM in Milli‐Q water adjusted to pH 2.0 or pH 8.0, and filtered (PTFE, 0.2 µm). 5 µl of this ThT preparation was added to 195 µl lysozyme amyloid fibril solution, 2 wt % at pH 2.0 and 0.4 wt % at pH 8.0, and mixed, before transferring to ibidi µ‐Slide glass bottom wells. FRAP experiments were performed using a Yokogawa W1 spinning disk confocal unit mounted on a Nikon Ti2 Eclipse inverted microscope. Imaging was carried out with a 100× Plan Apo oil immersion objective (NA 1.42). Fluorescence was detected using an Andor iXon Ultra EMCCD camera. A 405 nm laser was used for imaging (20%–30% power) and photobleaching (100% power). Emission was collected through a 450/50 nm band‐pass filter. Images were acquired with an exposure time of 250–400 ms. Three circular regions of interest (ROI) with a diameter of 3 µm were selected, inside a tactoid to be bleached (intensity *T*_1, *t*_), inside a nearby tactoid (*T*_2, *t*_), and in the background (*B_t_*). Photobleaching was performed in the first ROI with a dwell time of 200–1000 µs per pixel. Following bleaching, recovery was monitored at the three ROIs using NIS‐Elements (Nikon Instruments). For analysis, the intensity of the bleached ROI was normalized using $I_{t}=\frac{T_{1,t}-B_{t}}{T_{1,0}-B_{0}}/\frac{T_{2,t}-B_{t}}{T_{2,0}-B_{0}}$ [42], where t was time and 0 the initial time point, which was fitted using I_t_ =  A(1 − e^−t/τ^) + C [43], where τ was the characteristic recovery time, and A and C the constants. Half‐time of recovery and apparent diffusion coefficient were calculated using τ_(1/2)_ =  τ × ln 2 and D_app_ = (0.88ω^2^)/4τ_(1/2)_  respectively, where ω was the radius of ROI [44]. The mobile fraction was calculated from *I*_∞_ − *I*_bl_/*I*_0_ − *I*_bl_, where *I*_∞_, *I*_bl_ and *I*_0_ are the normalized intensities respectively after recovery, immediately after bleaching, and before bleaching.

### 5.8 Small‐Angle X‐Ray Scattering (SAXS) Measurements

SAXS measurements were conducted using either a Xenocs Xeuss 3.0 or a Bruker AXS Micro instrument, both equipped with copper (Cu) X‐ray source (λ  =  1.5418 Å, Cu Kα radiation). The Xenocs Xeuss 3.0 system delivered a beam size of 0.25×0.25 mm^2^. Sample‐to‐detector distance was set to 1.65 m. Scattered intensity was recorded using a Dectris EIGER2 detector with a beam stop‐free design, enabling absolute intensity measurements. In Bruker AXS Micro system (operated at 50 kV and 1000 µA), the beam was collimated using a 2D Kratky collimator, and the scattered intensity was collected using a 180° fully integrated 2D Pilatus 100K detector with a transparent primary beam‐stopper to enable absolute intensity measurements. Amyloid fibril solutions were loaded into 1.5 mm diameter cylindrical quartz capillaries (Hilgenberg) and sealed for vacuum measurements. The scattering vector was defined q  =  (4π/λ) sin θ, where 2θ was the scattering angle, and calibrated using silver behenate. 2D scattering was reduced using XSACT 2.6 (Xenocs) [45] for data collected with Xenocs Xeuss 3.0, and SAXSGUI [46] for data collected with Bruker AXS Micro. For each sample, 1D scattering profile of background (Milli‐Q water adjusted to the same pH as the sample) was subtracted from 1D scattering profile of sample. The resulting profiles were fitted to a cylindrical form factor using SasView 6.0.0 [47] when from an isotropic solution, or used to detect structure factor when from an isotropic–nematic coexistence solution.

### 5.9 Statistical Analysis

AFM data are presented as mean (± standard deviation) from analysis of hundreds of amyloid fibrils at each pH condition. Light scattering and electrophoretic mobility are representative of independent measurements. Charge density was best fit value (± 95% confidence intervals) from an independent measurement. The start concentration of LLCPS and the onset concentration of LLPS are from at least three independent measurements. SAXS and FRAP results are from a single sample preparation. Statistical analyses were conducted using Igor Pro (WaveMetrics) and MATLAB (MathWorks).

## Author Contributions

M.R. and R.M. designed the research. M.R. performed the experiments, analyzed the data, and drafted the original manuscript. M.R. and R.M. revised and approved the final manuscript.

## Conflicts of Interest

The authors declare no conflict of interest.

## Supporting information




**Supporting File 1**: advs74118‐sup‐0001‐SuppMat.pdf.

**Supporting File 2**: advs74118‐sup‐0002‐Video S1.mp4.

**Supporting File 3**: advs74118‐sup‐0003‐Video S2.mp4.

## Data Availability

The data that support the findings of this study are available from the corresponding author upon reasonable request.
