# Supplementary material for: Competition Between Liquid‐Liquid Crystalline Phase Separation (LLCPS) and Liquid‐Liquid Phase Separation (LLPS) in Amyloid Fibril Colloidal Systems
Source: Adv Sci (Weinh). 2026 Jan 28;13(19):e18781. doi: 10.1002/advs.202518781 (PMC13045426; doi:10.1002/advs.202518781)
Supplement: Supplementary file 1 — Supporting File 1: advs74118‐sup‐0001‐SuppMat.pdf. [file ADVS-13-e18781-s001.pdf]

Supporting Information for:

Competition Between Liquid-Liquid Crystalline Phase Separation (LLCPS) and Liquid-Liquid Phase Separation (LLPS) in Amyloid Fibril Colloidal Systems

Milad Radiom and Raffaele Mezzenga

Department of Health Sciences and Technology, ETH Zürich, Zürich 8092, Switzerland

Email: raffaele.mezzenga@hest.ethz.ch

Overview of Supporting Information

Complementary experimental data and theoretical modelling are provided in the following SI sections:

SI 1 presents supporting results for lysozyme amyloid fibrils.

Subsections in **SI 1**	Page
Hydrodynamic size and polydispersity index	3
Liquid crystalline domains at concentrations above LLCPS at pH 2.0	3
Fibril linear charge density	4
AFM characterization of lysozyme amyloid fibrils Lys-B	6
β-Sheet characterization of lysozyme amyloid fibrils as a function of pH using ThT assay	6
β-Sheet characterization of lysozyme amyloid fibrils as a function of pH using circular dichroism (CD)	7
Ion composition of dialyzed fibril samples from ICP-MS	8

SI 2 presents results for β-lactoglobulin amyloid fibrils.

Subsections in **SI 2**	Page
AFM characterization of β-lactoglobulin amyloid fibrils Blg-C	10
Hydrodynamic size and polydispersity index	11
LLCPS at pH 2.0	11
Fibril dynamics at pH 2.0	14
Inter-fibril arrangements at pH 2.0	14
pH-dependent phase transitions	15
pH-dependent inter-fibril arrangement	17
Fibril linear charge density	18
AFM characterization of β-lactoglobulin amyloid fibrils Blg-D	20

SI 3 presents the theoretical framework to extract fibril linear charge density.

Subsections in **SI 3**	Page
Theory	21
Fitting procedure	22
Consideration of counterion condensation	23
Sensitivity analysis	24

SI 4 (Page 30) provides theoretical calculations of the start concentration of LLCPS. **SI 5** (Page 33) presents the theoretical treatment of pair-interaction potential between fibrils. **SI 6** (Page 39) presents SI methods, including inductively coupled plasma mass spectrometry (ICP-MS), ThT assay and circular dichroism (CD).

SI 1. Chicken Egg White Lysozyme Amyloid Fibrils

Hereafter chicken egg white lysozyme (also called hen egg white lysozyme, HEWL) amyloid fibrils are referred to as "lysozyme" amyloid fibrils. Two independent lysozyme amyloid fibril samples were prepared, which were labelled Lys-A and Lys-B. AFM analysis gave L_c/D ~ 95 and 65, and L_p/L_c ~ 12 and 13, respectively for Lys-A and Lys-B.

A. Hydrodynamic size and polydispersity index

Hydrodynamic size (in terms of Z-average) and polydispersity index remained similarly "Before" and "After" electrophoretic mobility measurements across the pH range 2.0–12.0 (Figure SI 1-1).

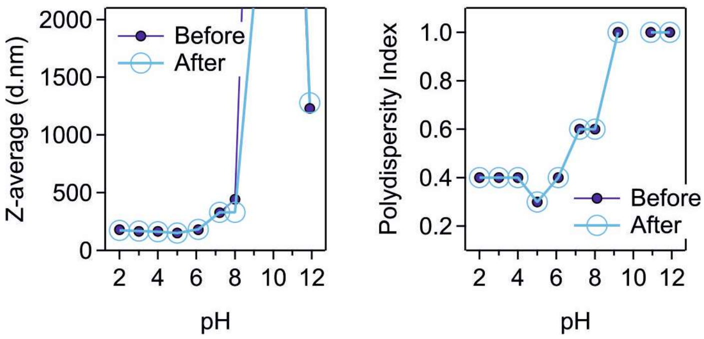

Figure SI 1-1: Z-average (left) and polydispersity index (right) of lysozyme amyloid fibrils Lys-A.

B. Liquid crystalline domains at concentrations above LLCPS at pH 2.0

Liquid crystalline domains at concentrations above the concentration series shown in Figure 2A (main text) were investigated using cross-polarized microscopy (Figure SI 1-2). The images were taken on day 3 post sample preparation. Complex liquid crystalline domains can be observed at all concentrations.

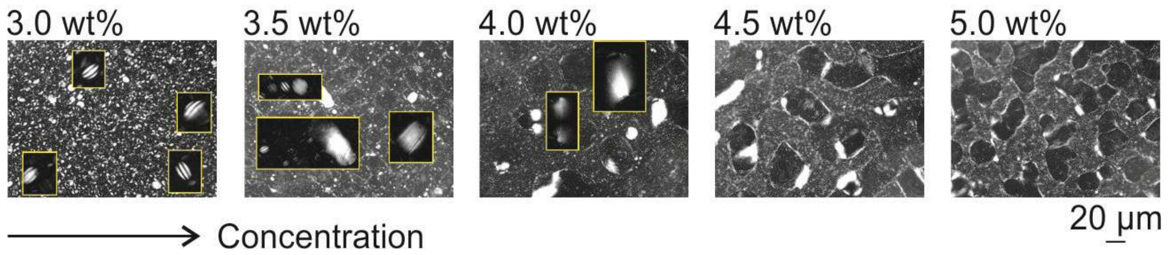

Figure SI 1-2: Cross-polarized microscopy images of lysozyme amyloid fibrils Lys-A at concentrations above the concentration series shown in Figure 2A (main text). Insets show regions of interest where cholesteric (3.0 wt %) or more complex nematic phases (3.5 to 5.0 wt %) could be identified. To improve imaging quality, microscopic samples were prepared using frames with 100 µm thickness.

C. Fibril linear charge density

The linear charge density of fibrils at each pH was obtained from the measurements of electrophoretic mobility and hydrodynamic size. The theoretical treatment of these data is presented in SI 3.

Figure SI 1-3 shows the hydrodynamic diameter (in terms of Z-average), polydispersity index, and electrophoretic mobility of fibrils at pH 2.0 as a function of ionic strength increments ΔI, adjusted by adding NaCl salt. From the distribution of hydrodynamic size versus ΔI, we found that the fibrils remained stable up to $\Delta I = 100\ \mathrm{mM}$. Thereby, a ΔI range from 0 to 100 mM was used to fit the electrophoretic mobility versus ΔI, according to the theory presented in SI 3, to obtain the best combination of fibril charge density and background ionic strength. The best fit parameters are shown in Table SI 1-1.

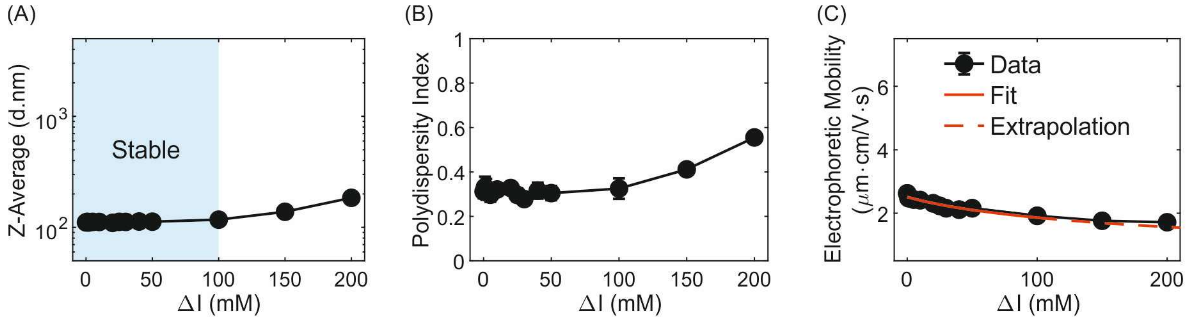

Figure SI 1-3: (A) Hydrodynamic diameter (in terms of Z-average), (B) polydispersity index, and (C) electrophoretic mobility of lysozyme amyloid fibrils Lys-B at pH 2.0 as a function of adjusted ionic strength ΔI. Electrophoretic mobility was fitted with the theoretical model in SI 3 (solid red line) within the stable ΔI range (shaded in panel (A)). Fit extrapolation to the outside of this range is shown with a dashed red line. Data are shown as mean ± standard deviation.

The same procedure was continued at pH 3.0 to 7.0, and the corresponding data and fits are presented in Figure SI 1-4. The best fit parameters for fibril charge density and background ionic strength at these pH values are reported in Table SI 1-1.

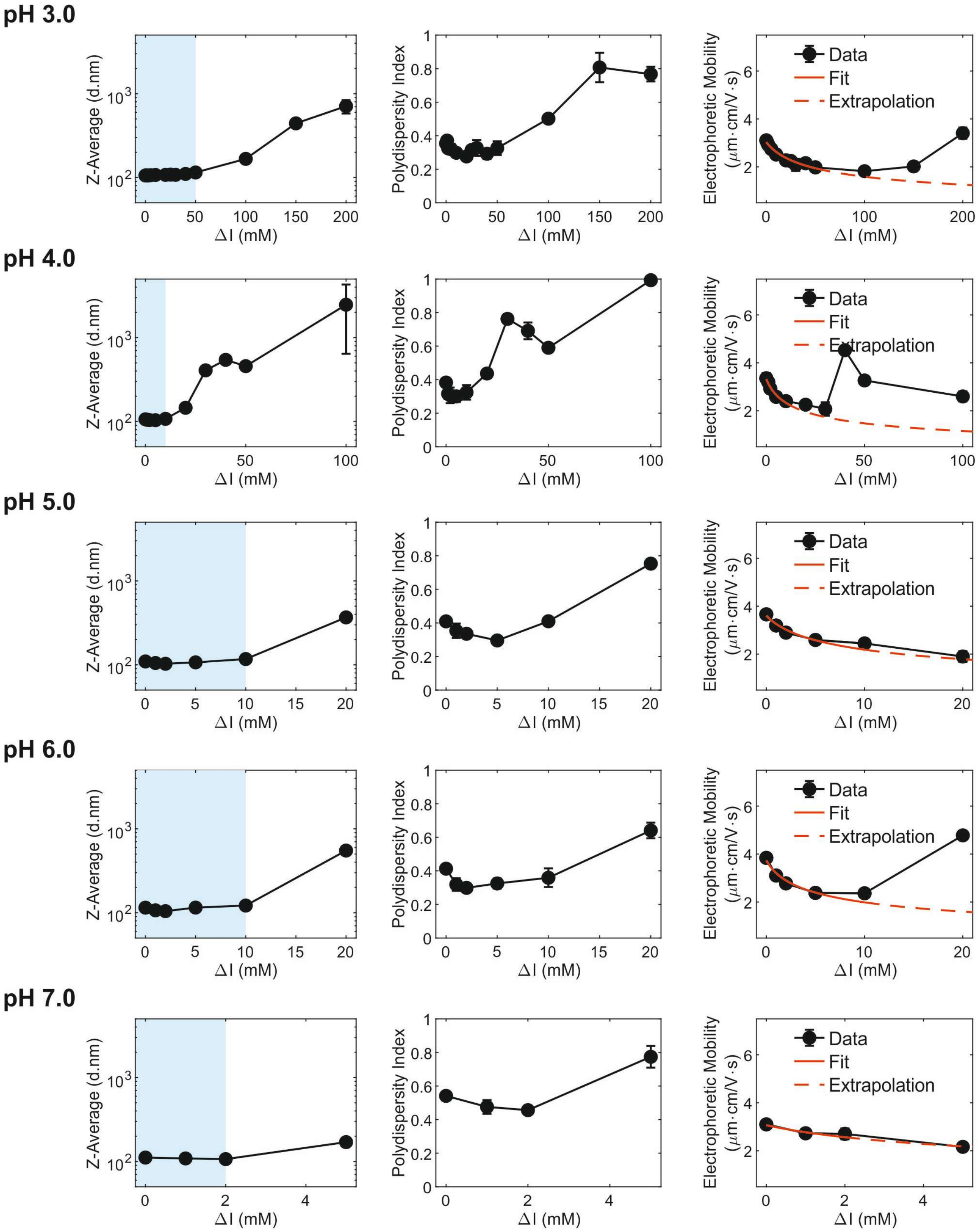

Figure SI 1-4: Hydrodynamic diameter (in terms of Z-average), polydispersity index, and electrophoretic mobility of lysozyme amyloid fibrils Lys-B at pH 3.0 to 7.0 as a function of ionic strength increments ΔI adjusted by NaCl. Each row corresponds to a specific pH, showing Z-average (left), polydispersity index (middle), and electrophoretic mobility (right). Electrophoretic mobility versus ΔI was fitted with the theoretical model in SI 3 (solid red line) within the stable ΔI range (shaded in panel (A)). Fit extrapolation to the outside of this range is shown with a dashed red line. Data are shown as mean ± standard deviation.

Table SI 1-1: The linear charge density λ of lysozyme amyloid fibrils and the residual ionic strength at each pH.

pH	λ (e/nm)	95% CI	I_o (mM)	95% CI
2.0	3.0	[2.8 3.2]	67.4	[49.6 85.3]
3.0	2.2	[2.1 2.3]	14.7	[11.0 18.3]
4.0	1.6	[1.4 1.7]	3.4	[1.8 4.9]
5.0	1.4	[1.2 1.5]	1.6	[0.7 2.4]
6.0	1.23	[1.07 1.39]	0.8	[0.1 1.5]
7.0	1.17	[0.91 1.43]	1.8	[0 3.7]

λ: linear charge density; I_o : residual or background ionic strength; CI: confidence interval

D. AFM characterization of lysozyme amyloid fibrils Lys-B

Figure SI 1-5 shows the AFM image of lysozyme amyloid fibrils Lys-B. Analysis of fibril geometry resulted in $L_c/D \sim 65$ and $L_p/L_c \sim 13$.

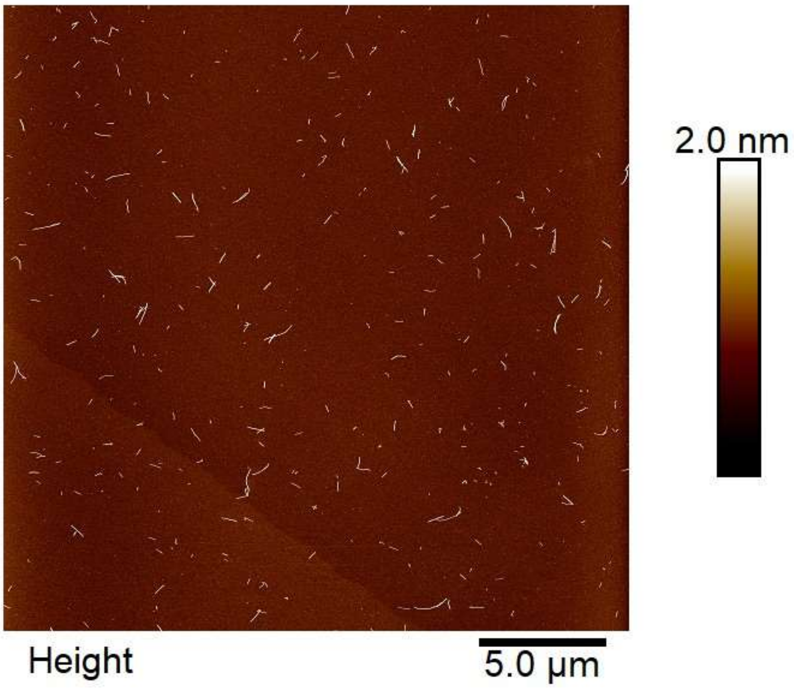

Figure SI 1-5: AFM image of lysozyme amyloid fibrils Lys-B at pH 2.0.

E. β-Sheet characterization of lysozyme amyloid fibrils as a function of pH using ThT assay

Lysozyme amyloid fibril Lys-B solutions at pH 2.0, 4.0, 6.0 and 8.0 were characterized using ThT assay. For pH 2.0, 4.0 and 6.0, samples were in the dilute single-fibril regime. At pH 8.0, an aliquot was collected from the middle fraction (see Figure 5B, main text); as a result, the concentration shown

on the x-axis for pH 8.0 overestimates the actual fibril concentration during the measurement. To account for pH-dependent effects on ThT fluorescence, background ThT signals were recorded at the corresponding pH values. Overall, similar ThT fluorescence profiles were observed across the entire pH range, indicating a comparable extent of β-sheet content in all samples. Therefore, pH adjustment did not affect the structural properties of the original amyloid fibrils prepared at pH 2.0.

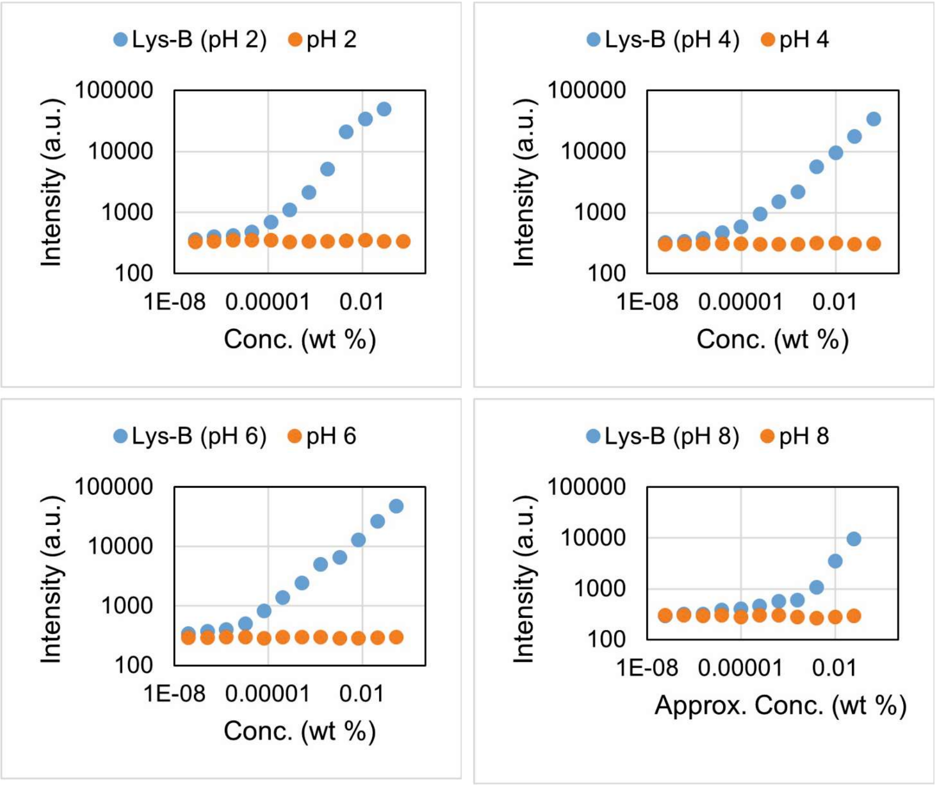

Figure SI 1-6: ThT fluorescence of lysozyme amyloid fibrils (Lys-B) at pH 2.0, 4.0, 6.0, and 8.0.

F. β-Sheet characterization of lysozyme amyloid fibrils as a function of pH using circular dichroism (CD)

CD measurements were performed on lysozyme amyloid fibril Lys-B samples at pH 2.0–8.0 to assess secondary-structure content across the conditions used in this study. The results are shown in Figure SI 1-7. In addition to five consecutive measurements and the mean spectrum (see Methods in **SI 6**), two vertical lines indicating the characteristic wavelengths associated with β-sheet structure (a positive peak at ~195 nm and a negative peak at ~215 nm) are shown. At pH 2.0, two measurements were collected: one labelled "High Salt", in which dilution to 0.025 wt % was performed directly at pH 2.0, and one labelled "Low Salt", in which the fibril solution at pH 2.0 (0.05 wt %) was diluted to 0.025 wt % in mQ water immediately before measurement. This approach minimized UV absorption effects from high ion concentrations (e.g., chloride at low pH). Under the "Low Salt" condition, the characteristic bands became evident. For pH 3.0 to 7.0, the spectra consistently exhibited the dominant

β-sheet signature, with well-defined positive and negative peaks (indicated by the vertical lines). At pH 8.0, the CD signal was diminished, likely by an increased scattering from dispersed LLPS condensates, which attenuated the CD intensity, although the general β-sheet features remained detectable. The CD spectra across the pH range demonstrated that the secondary structure of the fibrils remained β-sheet-rich and essentially unchanged. This result confirmed that the LLCPS/LLPS transitions observed in this work arise from differences in colloidal interactions and phase behavior rather than changes in the molecular structure of the fibrils.

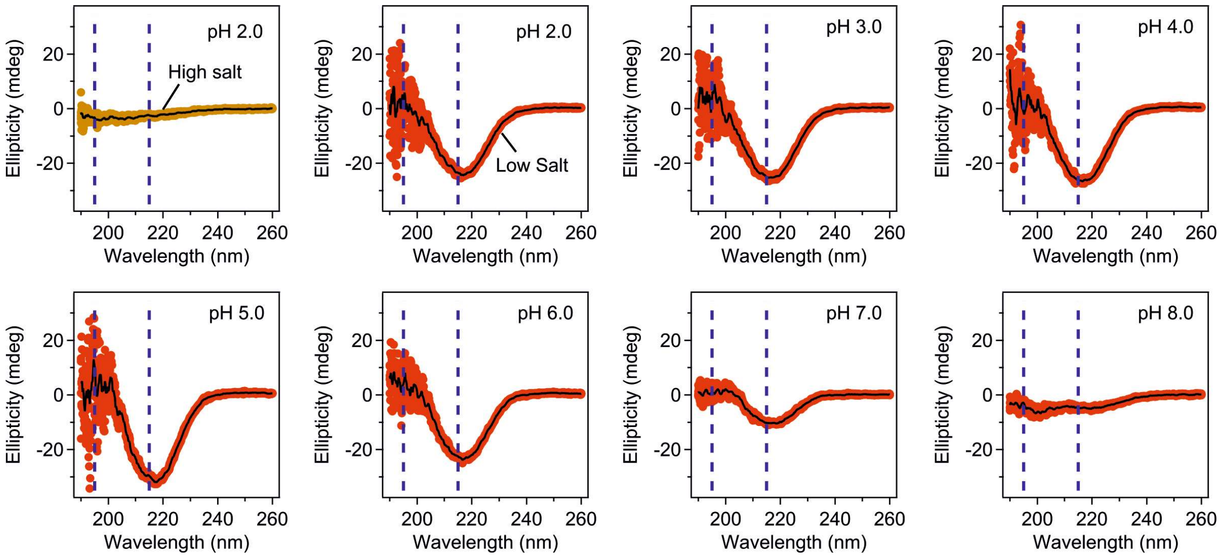

Figure SI 1-7: Circular dichroism (CD) spectra of lysozyme amyloid fibrils (Lys-B) across pH 2.0–8.0. Two vertical lines indicating the characteristic wavelengths associated with β-sheet structure, including a positive peak at ~195 nm and a negative peak at ~215 nm are shown in each panel. At pH 2.0, two different preparation protocols (high salt, and low salt) were performed (see text in SI).

G. Ion composition of dialyzed fibril samples from ICP-MS

We determined the ionic content of dialyzed lysozyme amyloid fibril Lys-B solutions by quantifying the concentrations of selected cations, including Na^{+}, K^{+}, Mg^{2+}, and Ca^{2+}, using inductively coupled plasma mass spectrometry (ICP-MS, see SI Methods). All samples were prepared by dialysis under the same conditions used for the phase behavior experiments. Cation concentrations were measured for fibril samples at pH 2.0 to pH 6.0. A PEG-containing sample at pH 2.0 (similar to the solution used in the reverse osmosis step for fibril up-concentration, see Methods in the main text) was also included.

Table SI 1-2 summarizes measured concentrations. Across all pH values, the dialyzed samples contained some levels of residual cations, with typical concentrations of Na^{+} in the range of 0.6–0.9 mM, K^{+} in the range of 0.045–0.066 mM, Mg^{2+} in the range of 0.001–0.003 mM, and Ca^{2+} in the range of 0.05–0.16 mM. The PEG control showed higher Na^{+} and Mg^{2+} concentrations, 12.7 mM and 0.014 mM respectively, but comparable K^{+} and Ca^{2+} concentrations, 0.074 mM and 0.17 mM respectively.

Table SI 1-2: Concentration of selected cations in solutions of lysozyme amyloid fibrils Lys-B at pH 2.0 to 6.0 and in a PEG solution (10 wt %) at pH 2.0.

**Cations**	**Na^+**			**Mg^{2+}**			**K^+**			**Ca^{2+}**		
**Sample**	**Conc. [mM]**	**SD [mM]**	**RSD [%]**	**Conc. [mM]**	**SD [mM]**	**RSD [%]**	**Conc. [mM]**	**SD [mM]**	**RSD [%]**	**Conc. [mM]**	**SD [mM]**	**RSD [%]**
**pH 2**	0.74	0.21	29.15	0.0018	0.0007	36.93	0.0489	0.0208	42.53	0.1095	0.0379	34.60
**pH 3**	0.92	0.12	13.39	0.0031	0.0021	67.73	0.0662	0.0012	1.75	0.1575	0.1262	52.48
**pH 4**	0.79	0.14	17.38	0.0019	0.0016	83.62	0.0497	0.0081	16.38	0.0979	0.1287	49.45
**pH 5**	0.82	0.19	23.19	0.0008	0.0008	96.54	0.0454	0.0071	15.58	0.0485	0.0374	38.55
**pH 6**	0.64	0.13	20.95	0.0011	0.0011	98.15	0.0602	0.0148	24.64	0.0634	0.0835	45.71
**PEG**	12.68	4.26	33.56	0.0140	0.0048	34.61	0.0742	0.0200	26.87	0.1657	0.0900	31.29

The ionic strength is calculated using:

$$I = \frac{1}{2}\sum z_i^2 c_i \quad (1)$$

which, for monovalent and divalent cations, reduces to:

$$\frac{1}{2}\left[\mathrm{Na}^+\right] + \frac{1}{2}\left[\mathrm{K}^+\right] + 2\left[\mathrm{Mg}^{2+}\right] + 2\left[\mathrm{Ca}^{2+}\right] \quad (2)$$

The ionic strength contribution of the cations in Table SI 1-2 at each pH are reported in Table SI 1-3.

Table SI 1-3: Ionic strengths contribution of solution cations at each pH calculated from Equation (2).

pH	Equation (2)
2.0	0.61 mM
3.0	0.81 mM
4.0	0.62 mM
5.0	0.53 mM
6.0	0.48 mM

The cations contribution to the ionic strength is similar, approximately 0.5–0.8 mM, in all samples, with only modest variation across pH. The calculated ionic strengths agree well with the minimum background (residual) ionic strengths extracted from electrophoretic mobility measurements (see Table SI 1-1) and the theoretical fitting of the phase boundaries (see Table SI 4-2). This confirms that pH and residual ionic strength were decoupled under the dialysis preparations in our work, supporting the assumption of a background ionic strength in the theoretical model of SI 4.

SI 2. β-Lactoglobulin Amyloid Fibrils

Investigation results using β-lactoglobulin amyloid fibrils are presented here. Four independent β-lactoglobulin amyloid fibril samples were prepared, which were labelled Blg-A, Blg-B, Blg-C and Blg-D. AFM analysis gave L_c/D ~ 150, 165, 80 and 62, and L_p/L_c ~ 12, 16, 17 and 15, respectively for Blg-A, Blg-B, Blg-C and Blg-D.

A. AFM characterization of β-lactoglobulin amyloid fibrils Blg-C

Figure SI 2-1 shows the AFM image and subsequent analysis of fibril height, contour length and persistence length of β-lactoglobulin amyloid fibrils Blg-C.

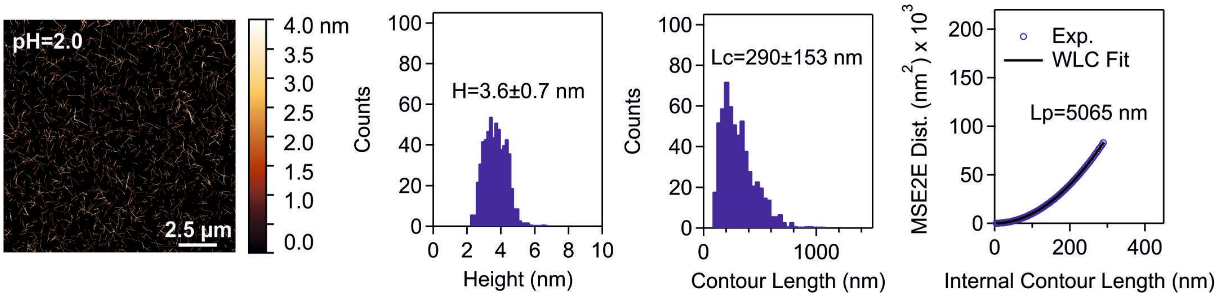

Figure SI 2-1: Representative AFM image of β-lactoglobulin amyloid fibrils Blg-C at pH 2.0 together with distributions of fibril height (H) and contour length (L_c). Persistence length (L_p) was calculated by fitting the mean squared end-to-end (MSE2E) distance of fibrils to worm-like chain (WLC) model (*1*). The ratio $L_p/L_c \sim 20$ indicates rigid rods. ± denotes standard deviation.

Figure SI 2-2 shows the AFM images of β-lactoglobulin amyloid fibrils Blg-C at pH 2.0, 3.0 and 4.0. Analysis of fibril geometry showed that they retained a rigid structure ($L_p/L_c \gg 1$) across the pH range 2.0 to 4.0.

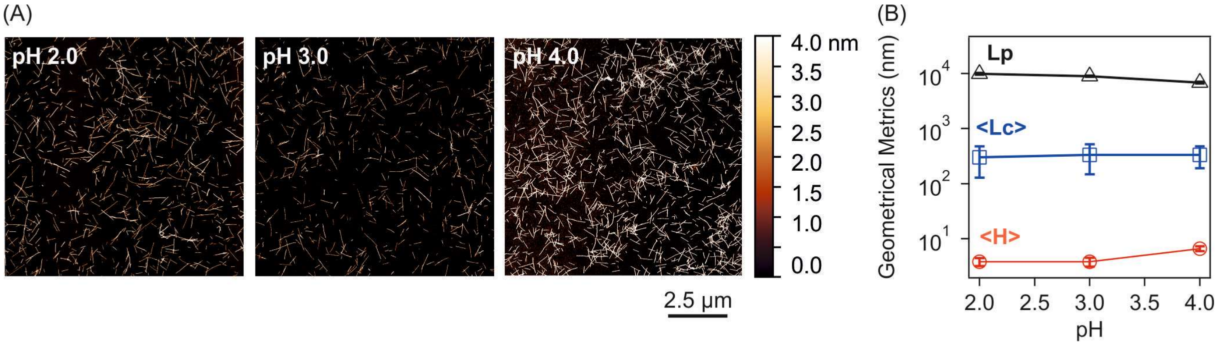

Figure SI 2-2: (A) Representative AFM images of β-lactoglobulin amyloid fibrils Blg-C at pH 2.0, 3.0, and 4.0, illustrating their preserved fibril features across this pH range. (B) Quantitative geometric

parameters, including fibril height (H), contour length (L_c), and persistence length (L_p). Error bar denotes standard deviation.

B. Hydrodynamic size and polydispersity index

Hydrodynamic size (in terms of Z-average) and polydispersity index remained similarly "Before" and "After" electrophoretic mobility measurements. From the electrophoretic mobility measurements, the isoelectric point of fibrils was found to be near pH 5.0.

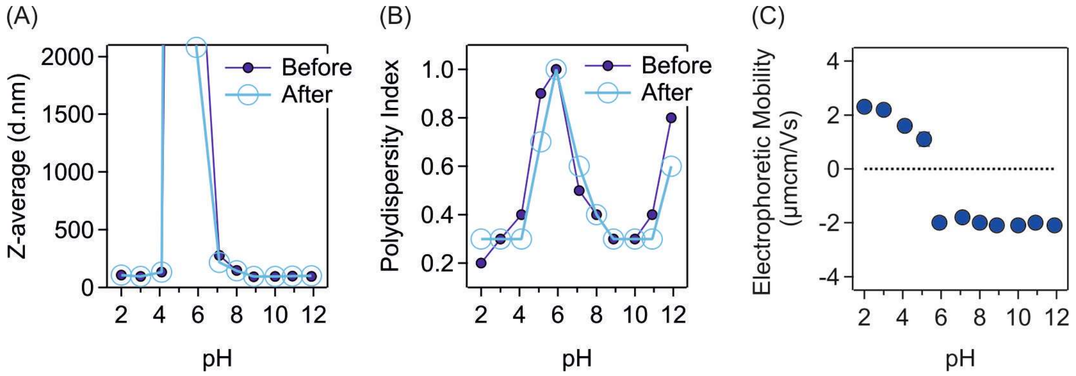

Figure SI 2-3: Hydrodynamic and electrophoretic properties of β-lactoglobulin amyloid fibrils Blg-C. (A, B) Hydrodynamic size (A) and polydispersity index (B) as a function of pH in the range 2.0 to 12.0, "Before" and "After" electrophoretic mobility measurements. (C) Electrophoretic mobility as a function of pH in the range 2.0 to 12.0.

C. LLCPS at pH 2.0

The LLCPS behavior of fibrils at pH 2.0 was investigated in samples Blg-A, Blg-B and Blg-C, prepared at different aspect ratios L_c/D . Blg-C had an aspect ratio of about 80 (Figure SI 2-1), while Blg-A and Blg-B had aspect ratios of about 150 (Figure SI 2-4) and 165 (Figure SI 2-5), respectively. After the start of LLCPS, distinct and large tactoids were observed in Blg-A and Blg-B solutions (Figure SI 2-4 and Figure SI 2-5), but not in Blg-C solutions (Figure SI 2-6). Nevertheless, as expected, liquid crystalline domains formed in Blg-C solutions after the start of LLCPS, which was assessed by measuring the standard deviation (STD) of grayscale intensity in the cross-polarized microscopy images. In particular, STD remained around 0 in isotropic solutions but showed a sharp increase above the start concentration of LLCPS.

The absence of large tactoids in Blg-C solutions is likely due to the smaller fibril aspect ratio (*2*) or a longer period of time to form large tactoids by these fibrils.

Fibrils of Blg-A and Blg-B transitioned from isotropic to isotropic-nematic coexistence at a concentration between 2.4–2.8 wt %, whereas fibrils of Blg-C transitioned between 4.8–6.0 wt %.

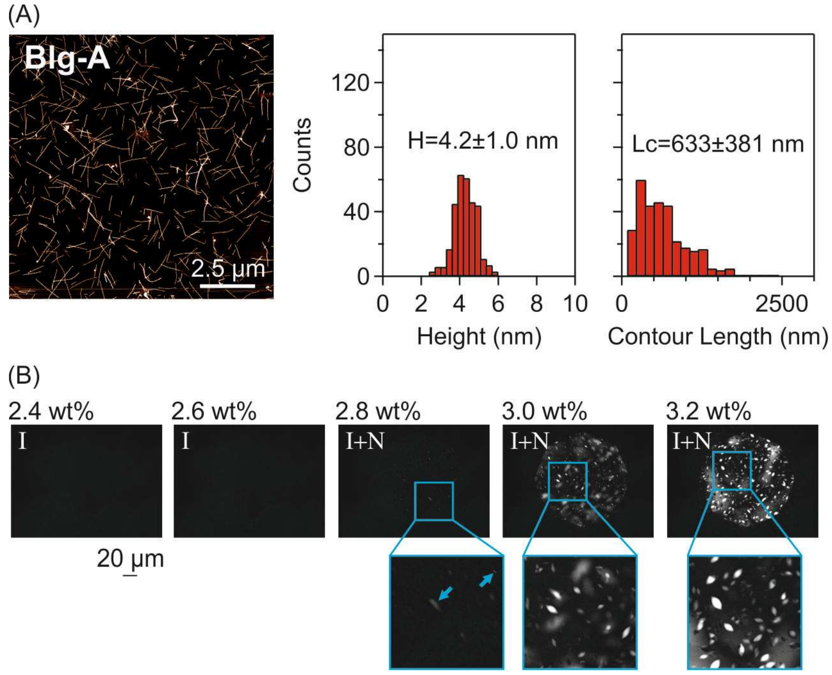

Figure SI 2-4: (A) Representative AFM image and distributions of fibril height (H) and contour length (Lc) of β-lactoglobulin amyloid fibrils Blg-A. (B) Representative cross-polarized microscopy images at pH 2.0, showing isotropic phase (I) at 2.4 and 2.6 wt % and isotropic-nematic coexistence (I+N) at 2.8, 3.0 and 3.2 wt %. Tactoids are visible in the microscopy images of I+N. Arrows in the inset at 2.8 wt % point at selected tactoids.

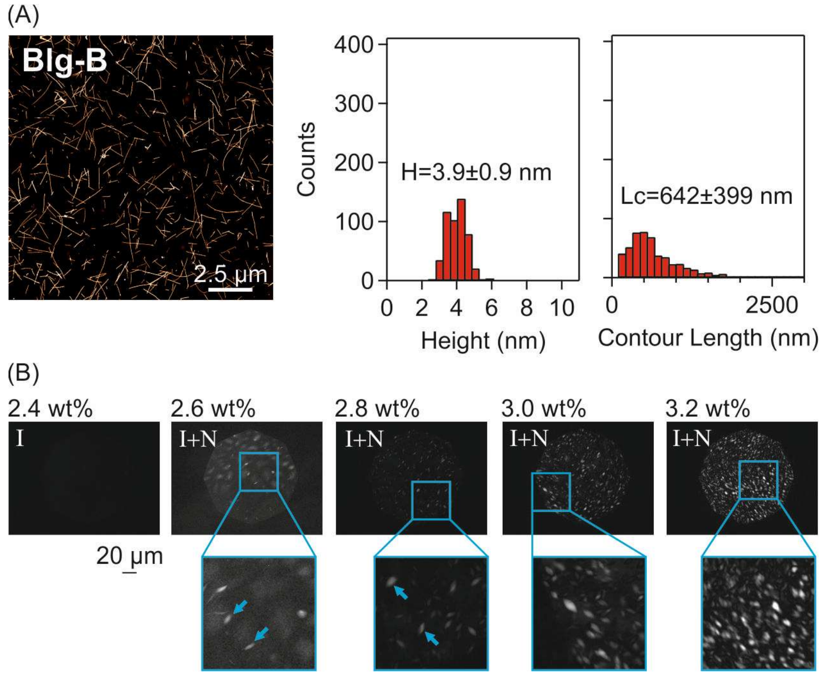

Figure SI 2-5: (A) Representative AFM image and distributions of fibril height (H) and contour length (Lc) of β-lactoglobulin amyloid fibrils Blg-B. (B) Representative cross-polarized microscopy images at pH 2.0, showing isotropic phase (I) at 2.4 wt % and isotropic-nematic coexistence (I+N) at 2.6, 2.8,

3.0 and 3.2 wt %. Tactoids are visible in the microscopy images of I+N. Arrows in the inset at 2.6 and 2.8 wt % point at selected tactoids. A different brightness and contrast setting was used for the inset image at 2.6 wt%.

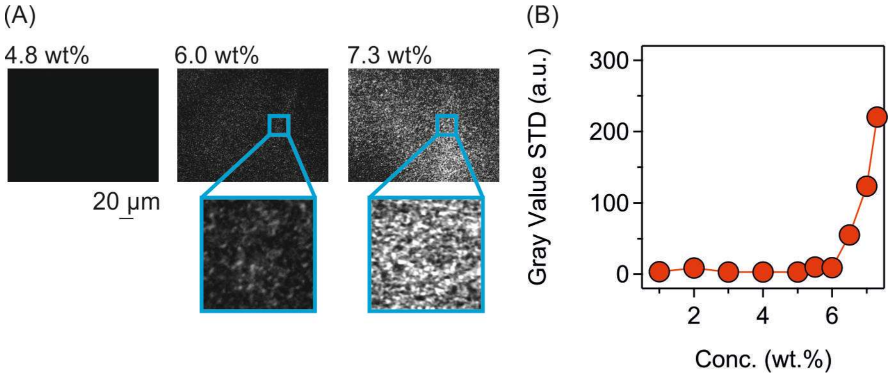

Figure SI 2-6: (A) Representative cross-polarized microscopy images of β-lactoglobulin amyloid fibrils Blg-C at pH 2.0, showing isotropic phase at 4.8 wt % and isotropic-nematic coexistence above 6.0 wt %. The insets show selected regions in isotropic-nematic coexistence highlighting the liquid crystalline domains. (B) The standard deviation (STD) in grayscale intensity as a function of concentration.

The liquid crystalline domains observed in Blg-C solutions at pH 2.0 and concentration 6.0 wt % exhibited periodic variation in grayscale intensity upon sample rotation confirming an angular dependence of birefringence in these domains (Figure SI 2-7). In particular, during rotation, the orientation of liquid crystalline domains change with respect to the fixed light polarization, leading to periodic variation of grayscale intensity.

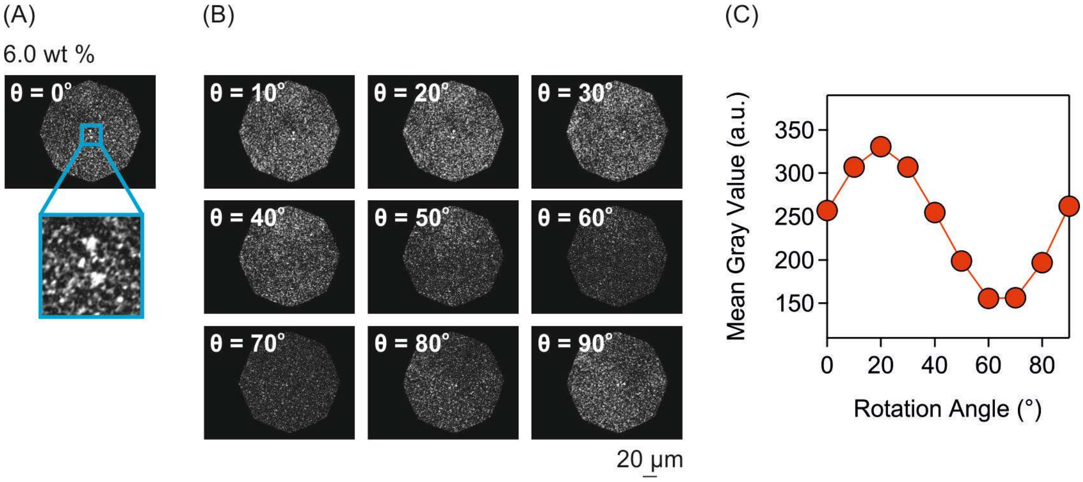

Figure SI 2-7: (A) Cross-polarized microscopy image of β-lactoglobulin amyloid fibrils Blg-C at pH 2.0 and concentration 6.0 wt %. The inset shows a selected region in the sample, highlighting liquid

crystalline domains. (B) Cross-polarized microscopy images of the same sample obtained by rotating it from θ = 0° (A) in 10° increments up to 90° with respect to the fixed positions of polarizer and analyzer. (C) Variation of mean grayscale intensity as a function of the sample rotation angle.

D. Fibril dynamics at pH 2.0

Fibril dynamics in isotropic and isotropic-nematic coexistence were investigated using 3D cross-correlation dynamic light scattering (Figure SI 2-8). From 0.05 to 3.0 wt %, a single diffusion coefficient, corresponding to the free diffusion of fibrils, was measured. From 4.0 to 7.0 wt % two distinct dynamic modes appeared; a fast mode with the same diffusion coefficient as measured at the lower concentrations, and a slower mode with about an order-of-magnitude lower diffusion coefficient. The latter is likely associated with the restricted fibril dynamics within the tactoids.

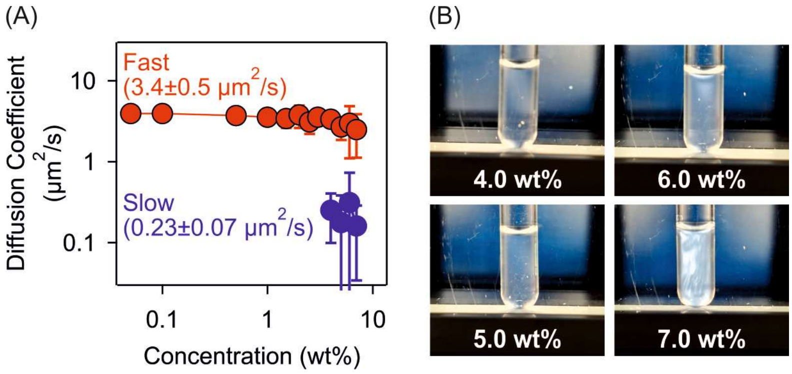

Figure SI 2-8: (A) Concentration-dependent diffusion coefficients ("Fast" and "Slow") of β-lactoglobulin amyloid fibrils Blg-C at pH 2.0 and across a concentration range 0.05–7.0 wt %. (B) Photographs of the fibril solutions at 4.0 wt % to 7.0 wt % between two crossed linear polarizers, showing the presence of birefringent regions from above 5.0 wt %.

E. Inter-fibril arrangements at pH 2.0

The fibril dimensions in isotropic solutions of Blg-C (1.0 to 4.8 wt %) were investigated using small angle X-ray scattering (SAXS). The intensity profiles versus momentum transfer q were consistent with form factor of rigid cylinders. Fitting yielded a fibril diameter of 2.8 nm and a length of 810 nm (Figure SI 2-9).

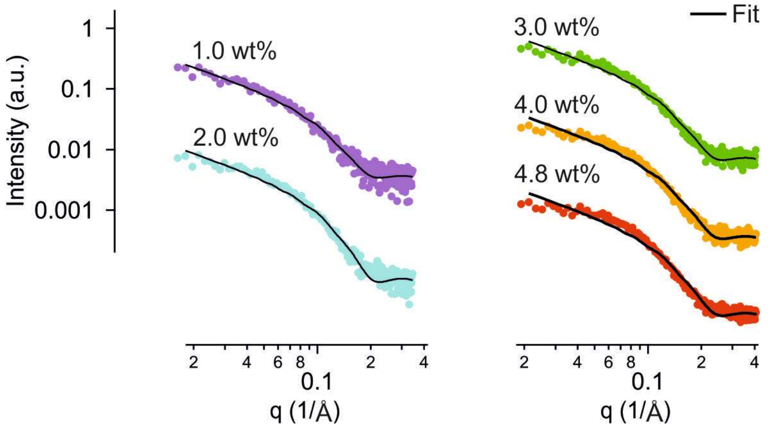

Figure SI 2-9: SAXS profiles of β-lactoglobulin amyloid fibrils Blg-C at pH 2.0 and at concentrations 1.0 to 4.8 wt.%. Data were fitted to cylindrical form factor.

F. pH-dependent phase transitions

Transition from LLCPS at pH 2.0 to LLPS at higher pH values was investigated. Photographs of samples under normal illumination showed transparency at pH 2.0 (7.3 wt %) and 3.0 (5.5 wt %), and translucency at pH 4.0 (4.4 wt %) and 5.0 (0.4 wt %) (Figure SI 2-10). Between two crossed linear polarizers, all solutions showed birefringence, either in form of large, extended birefringent domains, or in form of suspended microscopic birefringent condensates (Figure SI 2-10). In particular, structural colors were visible at pH 2.0 and 3.0, bulk birefringence lessened at pH 4.0 (coinciding with solution translucency), and at pH 5.0, microscopic birefringent condensates were visible.

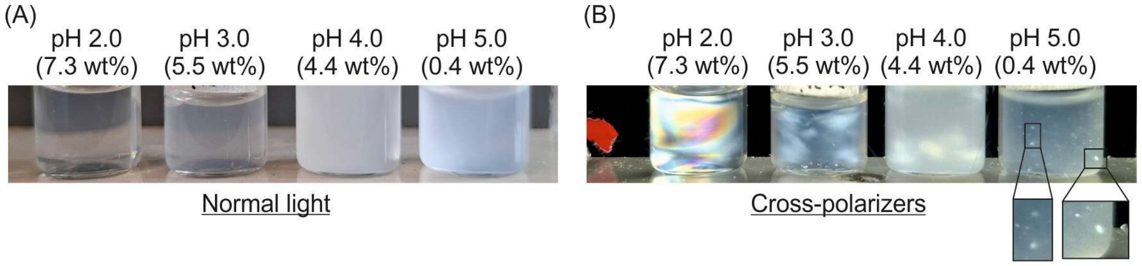

Figure SI 2-10: Photographs of β-lactoglobulin fibrils Blg-C solutions at different pH under normal light (A) and between two crossed linear polarizers (B). The inset at pH 5.0 shows microscopic birefringent condensates in the solution. Glass tube diameter = 1 cm.

The samples were then investigated using cross-polarized microscopy (Figure SI 2-11). At pH 3.0, a transition from isotropic to isotropic-nematic coexistence was observed between 4.0 and 5.0 wt %, which was further corroborated by the analysis of the standard deviation (STD) in the grayscale intensity versus concentration. At pH 4.0, the transition occurred between 2.5 and 3.5 wt %, but it was not as sharp as the transition at pH 3.0.

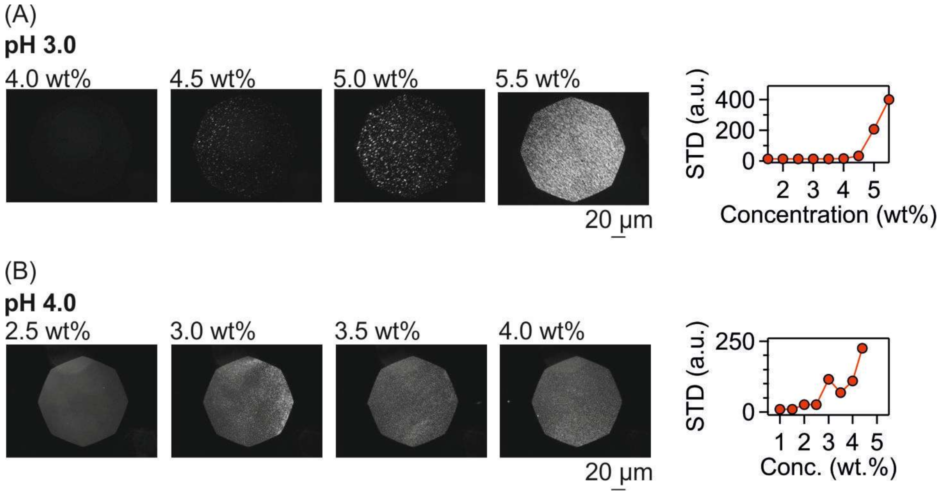

Figure SI 2-11: Cross-polarized microscopy images of β-lactoglobulin fibrils Blg-C solutions at pH 3.0 (A) and pH 4.0 (B), together with quantification of standard deviation (STD) of grayscale intensity as a function of concentration.

At pH 5.0, cross-polarized microscopy revealed the formation of large, irregular microscopic condensates, which contained multiple birefringent domains (Figure SI 2-12). These condensates lacked a well-defined nematic order (director). Dilution experiments while maintaining pH 5.0 revealed a moderate reversibility to the isotropic phase; however, returning the solution to acidic condition by diluting with pH 2.0 led to complete reversibility to the isotropic phase.

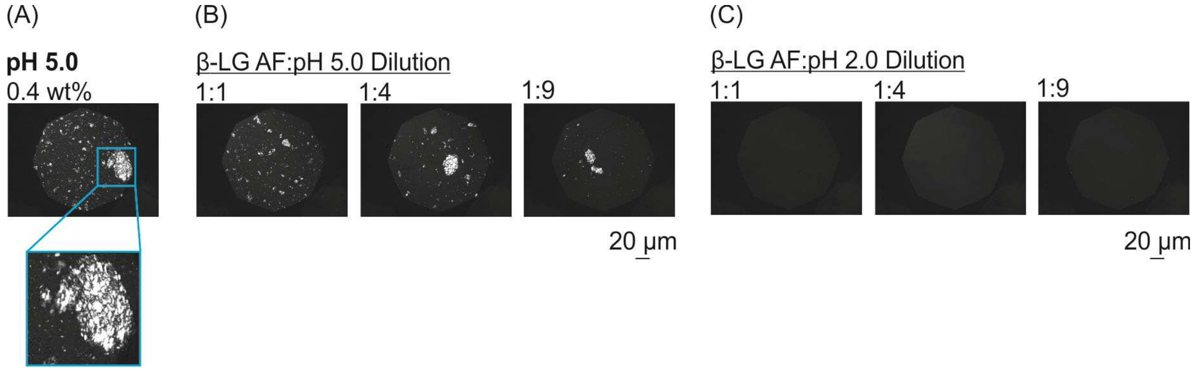

Figure SI 2-12: (A) Cross-polarized microscopy image of β-lactoglobulin fibrils Blg-C solution at pH 5.0 showing large and irregular clusters of birefringent domains at 0.4 wt %. (B, C) Moderate reversibility of phase separation upon dilution at pH 5.0 (dilution ratios 1:1 to 1:9) (B), and complete reversibility to the isotropic phase upon acidification by pH 2.0 (dilution ratios 1:1, 1:4 and 1:9) (C). β-LG AF:β-lactoglobulin amyloid fibril.

The condensates at pH 5.0 exhibited birefringent domains under cross-polarized microscopy. However, the objects lacked a coherent nematic order (director), regularly observed with nematic and chiral nematic (cholesteric) tactoids (*3*). The latter was further investigated by rotating a condensate at pH 5.0 against a fixed polarized light, as shown in Figure SI 2-13. Upon rotation, distinct birefringent domains in the condensate turn on and off.

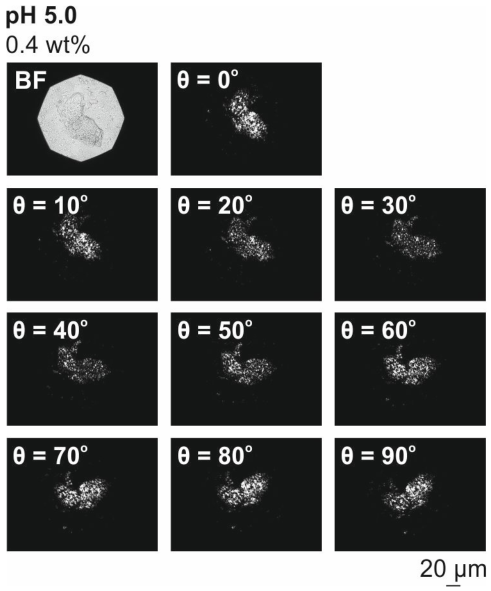

Figure SI 2-13: Bright field (BF) and cross-polarized microscopy images of β-lactoglobulin amyloid fibrils Blg-C at pH 5.0 and concentration 0.4 wt %. The cross-polarized microscopy images were obtained by rotating the sample from θ = 0° to 90° with respect to a fixed position of polarizer and analyzer.

From these experiments the critical concentration for LLCPS was found to decrease with pH, until near the isoelectric point of the fibrils where the system exhibited LLPS. The concentration versus pH phase diagram is shown in Figure SI 2-14.

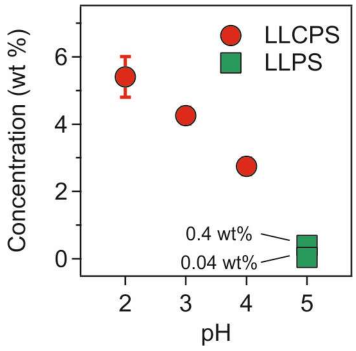

Figure SI 2-14: Phase diagram of concentration versus pH at the start of LLCPS and onset of LLPS, as determined by cross-polarized microscopy. The phase diagram is associated with the fibrils Blg-C.

G. pH-dependent inter-fibril arrangements

The pH-dependent inter-fibril arrangements were investigated using SAXS, and the results are shown in Figure SI 2-15. The profiles were almost identical at pH 2.0 and 3.0, suggesting consistent fibril structure (form factor). At pH 4.0 and at low q values, the intensity increased, suggesting attractive inter-fibril interactions which is consistent with the reduced charge density of the fibrils at pH 4.0. The profile at pH 5.0 was distinctly different.

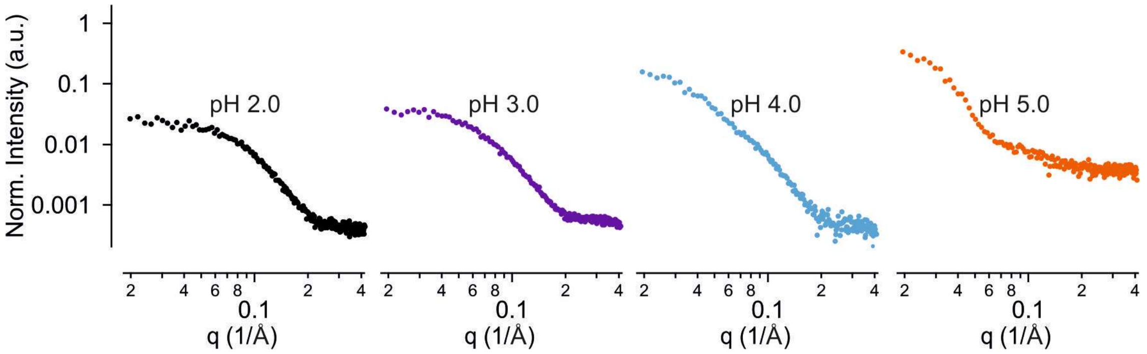

Figure SI 2-15: SAXS profiles of β-lactoglobulin amyloid fibrils Blg-C at pH 2.0 to 5.0.

H. Fibril linear charge density

The linear charge density of fibrils at each pH was obtained from the measurements of electrophoretic mobility and hydrodynamic size. The theoretical treatment of these data is presented in SI 3.

Figure SI 2-16 shows the hydrodynamic diameter (in terms of Z-average), polydispersity index, and electrophoretic mobility of fibrils at pH 2.0 and as a function of ionic strength increments ΔI, adjusted by adding NaCl salt. From the distribution hydrodynamic size versus ΔI, we found that the fibrils remained stable up to $\Delta I = 150$ mM at pH 2.0. Thereby, a ΔI range from 0 to 150 mM was also used to fit the electrophoretic mobility versus ΔI, according to the theory presented in SI 3, to obtain the best combination of fibril charge density and background ionic strength. The best fit parameters are shown in Table SI 2-1.

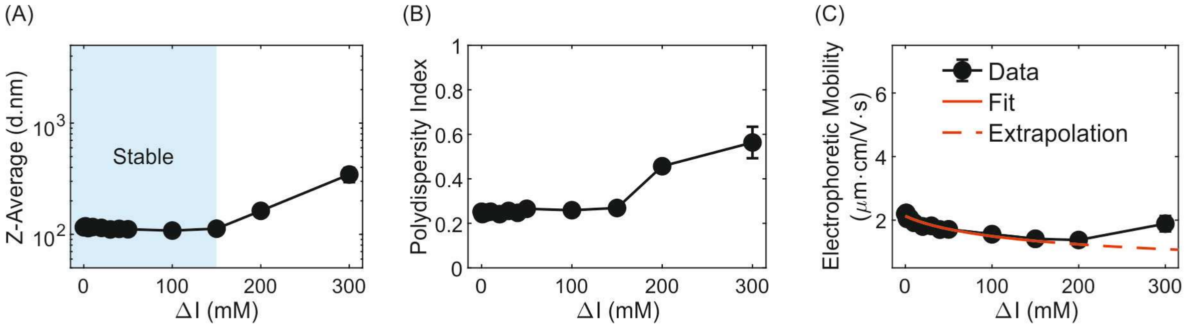

Figure SI 2-16: (A) Hydrodynamic diameter (in terms of Z-average), (B) polydispersity index, and (C) electrophoretic mobility of β-lactoglobulin amyloid fibrils Blg-D at pH 2.0 as a function of adjusted ionic strength ΔI. Electrophoretic mobility was fitted with the theoretical model in SI 3 (solid red line) within the stable ΔI range (shaded in panel (A)). Fit extrapolation to the outside of this range is shown with a dashed red line. Data are shown as mean ± standard deviation.

The same procedure was continued at pH 3.0 and 4.0, and the corresponding data and fits are presented in Figure SI 2-17. The best fit parameters for fibril charge density and background ionic strength at these pH values are reported in Table SI 2-1.

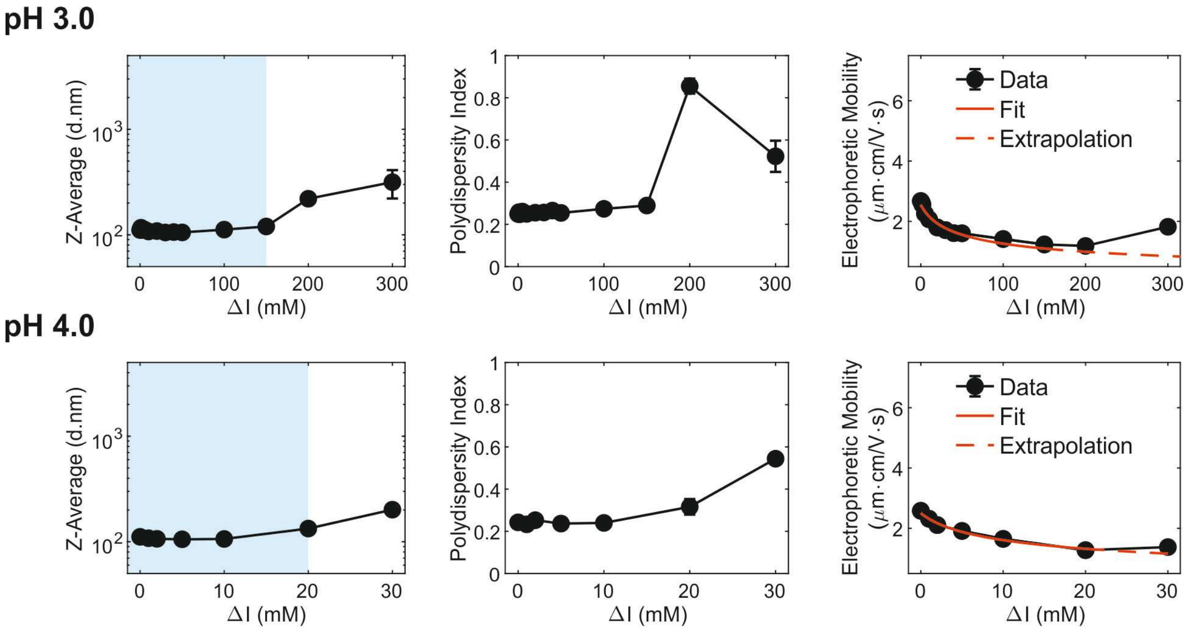

Figure SI 2-17: Hydrodynamic diameter (in terms of Z-average), polydispersity index, and electrophoretic mobility of β-lactoglobulin amyloid fibrils Blg-D at pH 3.0 and 4.0 as a function of ionic strength increments ΔI adjusted by NaCl. Each row corresponds to a specific pH, showing Z-average (left), polydispersity index (middle), and electrophoretic mobility (right). Electrophoretic mobility versus ΔI was fitted with the theoretical model in SI 3 (solid red line) within the stable ΔI range (shaded in panel (A)). Fit extrapolation to the outside of this range is shown with a dashed red line. Data are shown as mean ± standard deviation.

Table SI 2-1: The linear charge density λ of β-lactoglobulin amyloid fibrils and the residual ionic strength at each pH.

pH	λ (e/nm)	95% CI	I_o (mM)	95% CI
2.0	2.3	[2.1 2.5]	54.4	[36.3 72.5]
3.0	1.7	[1.6 1.8]	12.6	[7.7 17.4]
4.0	1.0	[0.9 1.1]	2.3	[1.4 3.2]

λ: linear charge density; I_o : residual or background ionic strength; CI: confidence interval

These measurements showed that at pH 2.0, the fibrils remained stable (single-fibril phase) at low to moderate ionic strength $I = I_o + \Delta I \leq 200$ mM , but at higher pH values 3.0 and 4.0, the stability

window became narrower, i.e., ≤ 160 mM at pH 3.0, and ≤ 20 mM at pH 4.0. The pH versus ionic strength phase diagram of the fibrils is presented in Figure SI 2-18. In addition, the linear charge density of the fibrils decreased progressively with increasing pH, from ~ 2.3 e/nm at pH 2.0 to nearly 1.0 e/nm at pH 4.0 (Figure SI 2-18). This reduction is consistent with approaching the isoelectric point of the fibrils near pH 5.0.

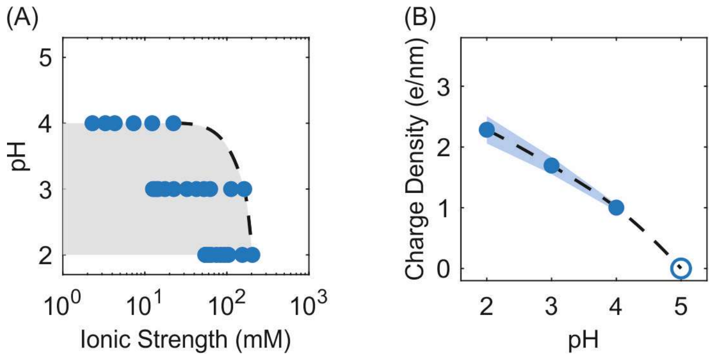

Figure SI 2-18: Phase behavior and linear charge density of β-lactoglobulin amyloid fibrils Blg-D. (A) Phase diagram showing pH versus ionic strength in a 1:1 electrolyte solution. Shaded area marks the region of stable, single fibril phase. (B) Fibril linear charge density as a function of pH. Shaded area represents the 95% confidence interval. Open circle at pH 5.0 denotes the isoelectric point. Dashed lines in (A) and (B) are guides to the eye.

I. AFM characterization of β-lactoglobulin amyloid fibrils Blg-D

Figure SI 2-19 shows the AFM image of β-lactoglobulin amyloid fibrils Blg-D. Analysis of fibril geometry resulted in $L_c/D \sim 62$ and $L_p/L_c \sim 15$.

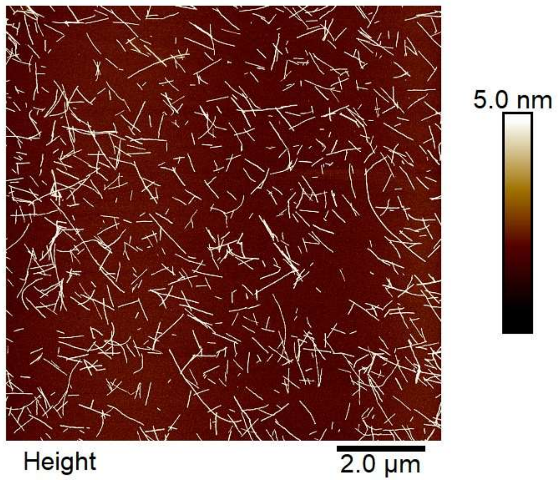

Figure SI 2-19: AFM image of β-lactoglobulin amyloid fibrils Blg-D at pH 2.0.

SI 3. Theoretical Calculations of Linear Charge Density

A. Theory

The orientationally averaged electrophoretic mobility μ_{av} of amyloid fibrils was determined using electrophoretic mobility measurements (see Methods in the main text). The pH of solutions, from the initial pH 2.0, was adjusted to a target pH (3.0 to 7.0 for lysozyme amyloid fibrils Lys-B, pH 3.0 and 4.0 for β-lactoglobulin amyloid fibrils Blg-D) using dialysis. Residual salt from sample preparation contributed to a background ionic strength I_{o} . From this background, the ionic strength was systematically varied by the addition of NaCl to reach a total ionic strength $I = I_{\mathrm{o}} + \Delta I$. Measurements were performed at a fixed pH and across a range of $\Delta I = [1 - 200]\,\mathrm{mM}$.

The experimentally determined electrophoretic mobility μ_{av} was related to zeta potential ζ using the generalized Henry function for infinitely long cylindrical colloidal particles (*4*):

$$\mu_{\mathrm{av}} = \frac{\varepsilon_{\mathrm{r}}\varepsilon_{\mathrm{o}}}{3\eta}\zeta\left[1 + 2f\left(\kappa a\right)\right] \tag{3}$$

where a is the cylinder radius (= 2 nm for fibrils), ε_{r} the relative permittivity (= 78.5 for water at 23°C), $\varepsilon_{\mathrm{o}} = 8.854 \times 10^{-12}\ \mathrm{F/m}$ the vacuum permittivity, η the solution viscosity (= 0.93 mPa s for water at 23°C), and κ the inverse Debye length which was determined from the total ionic strength (*5*):

$$\kappa = \left(\frac{2e^2 N_{\mathrm{A}} I}{\varepsilon_{\mathrm{r}}\varepsilon_{\mathrm{o}} kT}\right)^{1/2} \tag{4}$$

where $N_{\mathrm{A}} = 6.02 \times 10^{23}\ \mathrm{mol^{-1}}$ is the Avogadro's number, $kT = 4.09 \times 10^{-21}\ \mathrm{J}$ the thermal energy, and $e = 1.602 \times 10^{-19}\,\mathrm{C}$ the elementary charge. Rearranging Equation (3) gives:

$$\zeta = \frac{3\eta\mu_{\mathrm{av}}}{2\varepsilon_{\mathrm{r}}\varepsilon_{\mathrm{o}}}\left[\frac{1}{2} + f\left(\kappa a\right)\right]^{-1} \tag{5}$$

The analytical approximation for the Henry function $f\left(\kappa a\right)$ is then employed (*4*):

$$f\left(\kappa a\right) = \frac{1}{2}\left[1 + \left(1 + 2.55\big/\left[\kappa a\left\{1 + \exp\left(-\kappa a\right)\right\}\right]\right)^{-2}\right] \tag{6}$$

which when inserted into Equation (5) yields:

$$\zeta = \frac{3\eta\mu_{\mathrm{av}}}{2\varepsilon_{\mathrm{r}}\varepsilon_{\mathrm{o}}}\left[1+\frac{1}{2}\left(1+2.55/\left[\kappa a\{1+\exp(-\kappa a)\}\right]\right)^{-2}\right]^{-1} \tag{7}$$

Equation (7) relates the zeta potential ζ and the electrophoretic mobility μ_{av} as a function of the inverse Debye length κ , that is implicitly, as a function of the total ionic strength (Equation (4)).

The surface charge density σ of a cylindrical colloidal particle is related to its surface potential ψ_{s} by the nonlinear Poisson–Boltzmann formulation (*6*):

$$\sigma = \frac{2\varepsilon_{\mathrm{r}}\varepsilon_{\mathrm{o}}\kappa kT}{e}\sinh\left(e\psi_{\mathrm{s}}/2kT\right)\left[1+\frac{K_1^2(\kappa a)/K_0^2(\kappa a)-1}{\cosh^2\left(e\psi_{\mathrm{s}}/4kT\right)}\right]^{1/2} \tag{8}$$

where $K_0(\kappa a)$ and $K_1(\kappa a)$ are the zeroth-order and first-order modified Bessel functions of the second kind. By approximating the surface potential by the zeta potential, i.e. $\psi_{\mathrm{s}} \approx \zeta$, Equations (7) and (8) provide a direct relationship between experimentally measured electrophoretic mobility μ_{av} and surface charge density σ. The linear charge density is finally related to surface charge density via:

$$\lambda = \frac{2\pi a}{e}\sigma \tag{9}$$

We experimentally measure μ_{av} as a function of ΔI , from which, by employing the theory presented above, we calculate the best combination of (σ, I_0) . In particular, for any trial values of (σ, I_0) , we numerically calculate the surface potential ψ_{s} as a function of the total ionic strength $I = I_{\mathrm{o}} + \Delta I$ (where ΔI is known experimentally from the added NaCl salt) by relying on Equations (4) and (8). From the distribution of ψ_{s} versus I, and under the assumption $\psi_{\mathrm{s}} \approx \zeta$, the theoretical electrophoretic mobility μ_{av} is calculated as a function of I via Equations (7) for the same trial values of (σ, I_0) . The final search for the best (σ, I_0) combination is detailed below.

B. Fitting procedure

The fitting of experimental electrophoretic mobility μ_{av} versus ΔI (c.f. Figure SI 1-3 and Figure SI 1-4 for lysozyme amyloid fibrils, and Figure SI 2-16 and Figure SI 2-17 for β-lactoglobulin amyloid fibrils) was performed using a custom MATLAB code with the following workflow at each pH:

(1) Electrophoretic mobility μ_{av} and Z-average were grouped by ionic strength increments ΔI , replicates were averaged, and standard deviations computed. 12 replicates for μ_{av} , and 5 replicates for Z-average were used.

(2) Z-average data were used to exclude ΔI conditions where aggregation occurred. We used an increase of more than 20% in Z-average relative to its value at $\Delta I = 0$ as the criterion of aggregation.

(3) Then, as a function of ΔI, surface potential ψ_s was calculated first by numerically solving Equation (8), given a trial σ and I_0. Equation (4) relates the ionic strength I to the inverse Debye length κ.

(4) Using the assumption mentioned earlier ($\psi_s \approx \zeta$), the electrophoretic mobility μ_{av} was then calculated from Equation (7).

At this stage, we have predicted electrophoretic mobility as a function of ΔI for an initial trial combinations of σ and I_0, and we are ready to contrast these values against the experimental values of mobility μ_{av} versus ΔI.

(5) Using nonlinear least-squares minimization with bounds on $\sigma =$ [0, 0.13] C/m^2 and $I_0 =$ [0, 100] mM, we find the best combination of these two parameters. At the end, we calculated the linear charge density from the final (best) value of the surface charge density using Equation (9). Confidence intervals (95%) were obtained from the Jacobian of the residual function. See Table SI 1-1 for lysozyme amyloid fibrils, and Table SI 2-1 for β-lactoglobulin amyloid fibrils.

C. Consideration of counterion condensation

We note that counterion condensation is not explicitly included in the equations above, for the following reason. Theoretically, when the charge density of fibrils exceeds a critical value such that the electrostatic potential energy of fibril–counterion interaction becomes greater than the thermal energy, counterions condense onto the charged fibril surface. The critical condition is described by Manning threshold, which is defined as:

$$(Q\lambda)/e > 1 \quad (10)$$

where $Q = e^2/(4\pi\varepsilon_r\varepsilon_0 kT)$ is the Bjerrum length. In water at 23°C, the Bjerrum length is $Q = 0.7$ nm. Therefore, the critical condition corresponds to linear charge densities greater than 1.4 e/nm.

In principle, to incorporate Manning condensation into the calculations above, one could readjust the linear charge density to 1.4 e/nm whenever the calculated values of λ exceed this threshold. For example, such a correction would apply to lysozyme amyloid fibrils at all pH values below 5.0 and to β-lactoglobulin amyloid fibrils at all pH values below approximately 4.0. However, the markedly different LLCPS and electrokinetic properties of fibrils at these pH values do not justify applying this

adjustment. For this reason, Manning condensation theory was not included in the theoretical interpretation of our results.

We finally note that the use of electrophoretic mobility to estimate the charge density inherently reflects the effective charge of the fibrils (including condensation effects). Electrophoretic measurements capture the motion of a colloidal particle under an applied field governed by zeta potential at the slipping plane and the hydrodynamics. As a result, any charge regulation arising from Manning condensation is implicit in the measured mobility. Consequently, our results do not necessarily indicate the absence of counterion condensation but rather this phenomenon is masked within the effective charge probed experimentally.

D. Sensitivity analysis

To assess the robustness of the linear charge density λ determined from electrophoretic mobility fits, we performed a sensitivity analysis aimed at quantifying how this parameter varied when selected model assumptions and physical parameters were altered within plausible ranges.

We used the electrophoretic mobility data of lysozyme amyloid fibril Lys-B at pH 2.0 for the sensitivity analysis. Under the default model fit (fibril radius $a = 2$ nm, freely optimized background ionic strength I_o, and Z-average filtering ratio $r = 1.2$), we obtained the values reported earlier and reiterated in Table SI 3-1.

Table SI 3-1: The estimated linear charge density λ of lysozyme amyloid fibrils Lys-B at pH 2.0 using the default model fit.

pH	λ (e/nm)	95% CI
2.0	3.0	[2.8 3.2]

For each altered parameter, the nonlinear least-squares fitting procedure described above was computed using identical convergence criteria. The resulting best-fit λ together with 95 % confidence intervals were then compared to the default fit value.

Fibril radius. Uncertainty in fibril radius is due to the presence of fibrils with varying number of filaments twisted into a mature fibril (*7*). We used AFM to measure fibril height H, and estimated fibril radius by $a = H/2 = 1.8 \pm 0.3$ nm (c.f. Figure 1B in the main text). Subsequently $a = 2$ nm was used as the default value, and in below we quantified variations in the best-fit λ values considering 10% uncertainty in a (Table SI 3-2).

Table SI 3-2: Best fit values for λ and I_o as a function of fibril radius $a = 2$ nm $\pm$ 10% uncertainty. Mobility fits (right column) display experimental data (black circles) with best-fit predictions (solid red) and extrapolations beyond the fitted range (dashed red).

a (nm)	λ (e/nm)	95% CI	I_o (mM)	95% CI	Mobility fits plot
1.8	2.8	[2.5, 3.0]	66.7	[48.9 84.5]	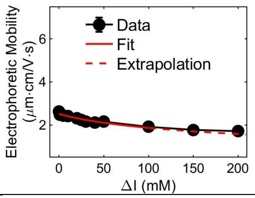
2.0	3.0	[2.8, 3.2]	67.4	[49.6 85.3]	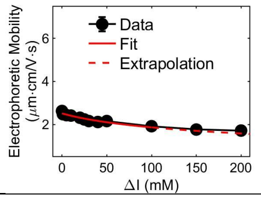
2.2	3.2	[3.0, 3.5]	68.0	[50.1 85.9]	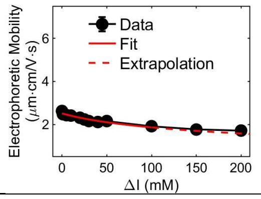

Table SI 3-2 shows that 10% uncertainty in fibril radius resulted in about 7% deviation in λ and about 1% deviation in I_o from their best fit values using the default fibril radius. We note however that these deviations are within the 95% confidence intervals of the default fit values.

We further varied a to 1.0 nm (corresponding to single-filament fibrils), 3.0 nm (three-filament fibrils) and 4.0 nm (four-filament fibrils). Each radius was then used to refit λ and I_o to the same data set, and Table SI 3-3 shows the resulting best-fit λ and I_o values, which in this case showed significant deviations from with the default fit values.

Table SI 3-3: Best fit values for λ and I_o for various values of a. Mobility fits (right column) display experimental data (black circles) with best-fit predictions (solid red) and extrapolations beyond the fitted range (dashed red).

a (nm)	λ (e/nm)	95% CI	I_o (mM)	95% CI	Mobility fits plot
1.0	1.8	[1.6, 1.9]	60.0	[43.2 76.8]	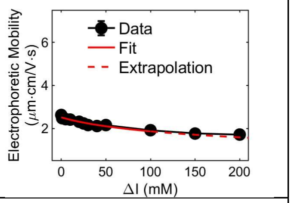
3.0	4.1	[3.8, 4.4]	69.6	[51.4 87.8]	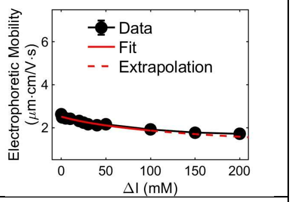
4.0	5.2	[4.7, 5.6]	71.4	[52.4 90.4]	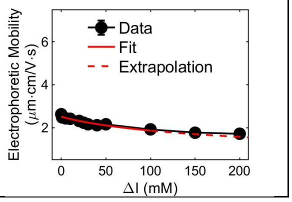

We conclude that precision in fibril radius estimation is important for an accurate evaluation of fibril charge density using our model. We note that AFM imaging in soft tapping mode is well suited to obtain fibril radius.

Background ionic strength. The baseline model fits I_o together with λ as a free parameter. To estimate the effect of uncertainties in I_o on the calculation of λ, we fixed I_o to its best fit value ± 20% uncertainty (Table SI 3-4).

Table SI 3-4: Best fit value for λ as a function of fixed I_o values. I_o was equated to its best fit value (67.4 mM) ± 20% uncertainty. Mobility fits (right column) display experimental data (black circles) with best-fit predictions (solid red) and extrapolations beyond the fitted range (dashed red).

I_o (mM)	λ (e/nm)	95% CI	Mobility fits plot
53.9	2.8	[2.75 2.86]	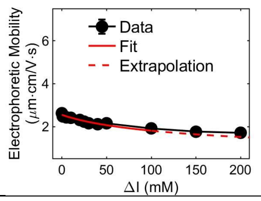
67.4	3.0	[2.8 3.2]	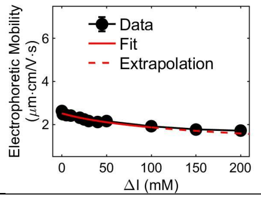
80.9	3.2	[3.11 3.22]	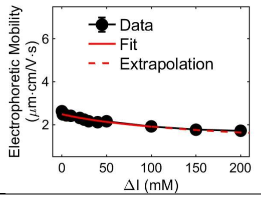

Table SI 3-4 shows that 20% uncertainty in the best fit value of I_o results in about 7% deviations from the best fit value of λ computed using the default values. These deviations are within the 95% confidence intervals of the default fit value of λ.

In the next step, we fixed I_o to several representative values, one corresponding to the solution pH, and the other equal to an extreme value of 150 mM.

Table SI 3-5: Best fit value for λ as a function of fixed I_o values. Mobility fits (right column) display experimental data (black circles) with best-fit predictions (solid red) and extrapolations beyond the fitted range (dashed red). Shaded rows highlight 20% deviation from the best fit value 67.4 mM.

I_o (mM)	λ (e/nm)	95% CI	Mobility fits plot
10 (pH 2.0)	1.9	[1.7 2.1]	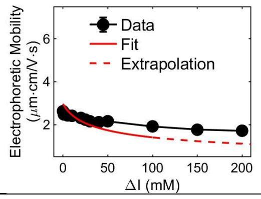
150	3.9	[3.7 4.0]	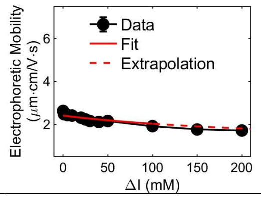

We conclude that both extreme values for I_o, one estimated from the solution pH (that is disregarding residual or background ionic strength, c.f. Discussion in the main text, and SI 4), and the other fixed at 150 mM result in large deviations of λ from its default fit value (3.0 e/nm). We note that 150 mM is straightforward to exclude from any fit due to the high ionic strength. However, the background ionic strength is more subtle. Interestingly the fit of the model to electrophoretic mobility as a function of ΔI showed noticeable deviations from the experimental data points if the background ionic strength was excluded from the fits (see 1st row in Table SI 3-5). The reader is referred to Table SI 1-2 for information regarding residual cations in solution of lysozyme amyloid fibrils at different pH values.

Z-average filtering ratio. The default model employs a selection criterion based on hydrodynamic size, $\text{Z-Average}_{\Delta I} < r \times \text{Z-Average}_{\Delta I=0}$ with $r=1.2$, to retain selected electrophoretic mobility points for fitting in the single-fibril phase. To evaluate the effect of this control threshold, r was varied from 1.1 to 1.3, and an "unfiltered" case (no exclusion) was also tested.

Table SI 3-6: Best fit values for λ and I_o for various values of the Z-average filtering ratio r. Mobility fits (right column) display experimental data (black circles) with best-fit predictions (solid red) and extrapolations beyond the fitted range (dashed red).

r	λ (e/nm)	95% CI	I_o (mM)	95% CI	Mobility fits plot
1.1	3.0	[2.8, 3.2]	67.4	[49.6 85.3]	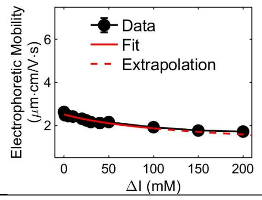
1.2	3.0	[2.8, 3.2]	67.4	[49.6 85.3]	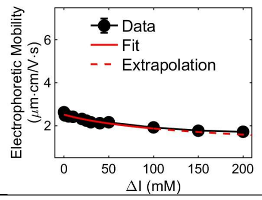
1.3	3.1	[2.9, 3.3]	72.5	[56.3 88.7]	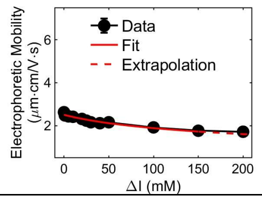
∞	3.1	[2.9, 3.4]	78.9	[61.8 96.1]	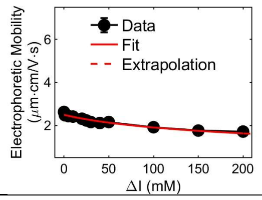

We find that the filtering ratio did not have a noticeable effect on the fitted values of λ (maximum 3% deviation from the default fit for $r=1.3$ and $r=\infty$), but showed noticeable variations to the best fit I_o value (8% deviation at $r=1.3$ and 17% deviation at $r=\infty$). Nevertheless we expect that the deviation from the best fit value of λ will become significant at the higher pH values.

Note relevant to the calculations presented in SI 4. The procedure described above allowed us to quantify the linear charge density as a function of pH, $\lambda(\mathrm{pH})$. In SI 4, this linear charge density is used to match the experimental and theoretical start concentration of LLCPS through Odijk's correction to Onsager's theory (*8, 9*). Specifically, by inserting the known values of the linear charge density at each pH into the theoretical start concentration of LLCPS, we extracted the in-solution (total) ionic strength I_{in} from the experimental start concentration of LLCPS.

SI 4. Theoretical Calculation of the Start Concentration of LLCPS

To estimate the start concentration of LLCPS, we proceeded as follows. The fibril volume fraction at the start of LLCPS was calculated according to (*8, 9*):

$$\phi_{\text{I-N}} = c^{*}\left(1-0.75h\right)^{-1}\left(\frac{D^{2}}{LD_{\text{eff}}}\right) \tag{11}$$

in which the equation parameters have the same definition as stated in the main text, and as given in SI 3. The effective diameter was calculated from (*8, 9*):

$$D_{\text{eff}} = D + \kappa^{-1}\left(\ln A' + C_{\text{E}} + \ln 2 - 0.5\right) \tag{12}$$

and the twisting factor from (*10*):

$$h = \left(\kappa D_{\text{eff}}\right)^{-1} \tag{13}$$

where κ^{-1} is the Debye length (see Equation (4)), A' the electrostatic interaction amplitude, and $C_{\text{E}} = 0.577$ the Euler's constant. The Debye-Hückel approximation gives for A' (*10*):

$$A' = \frac{8\pi\lambda^{2}Qe^{-\kappa D}}{\kappa^{3}D^{2}K_{1}^{2}\left(\kappa D/2\right)} \tag{14}$$

where $K_{1}\left(\kappa D/2\right)$ is the modified Bessel function of the second kind, and $Q = 0.7\ \text{nm}$ the Bjerrum length. Under our experimental conditions, the prefactor c^{*} in Equation (11) is equal to 3.659 (*2, 8, 9*).

To compare with experimental observations of the start of LLCPS, the fibril volume fraction was converted to weight concentration using:

$$c_{\text{I-N}}\left(\text{wt }\%\right) = \frac{\phi_{\text{I-N}}\rho_{\text{AF}}}{\phi_{\text{I-N}}\rho_{\text{AF}} + \left(1-\phi_{\text{I-N}}\right)\rho_{\text{W}}} \tag{15}$$

where $\rho_{\text{AF}} = 1.3\ \text{g/ml}$ and $\rho_{\text{W}} = 1.0\ \text{g/ml}$ are the fibril and water densities (*11*).

The Debye length κ^{-1} can be calculated conservatively using the nominal ionic strength:

$$I_{\text{nom}} = \frac{1}{2}\sum z_{\text{i}}^{2}c_{\text{i}} \tag{16}$$

where $z_i^2 = 1$ for the ionic species H^+, OH^-, Na^+, Cl^-, and c_i determined from pH.

Alternatively, κ^{-1} can be calculated from in-solution ionic strength:

$$I_{in} = I_o + I_{nom} \tag{17}$$

where I_o is resulting from residual (background) ionic strength due to Donnan effect (*12*).

We focus on lysozyme amyloid fibrils for the following calculations:

(I) Computed parameters, including A', D_{eff}, h, and $c_{I\text{-}N}$, using $\kappa^{-1}(I_{nom})$ are reported in Table SI 4-1. We note that while the theoretical calculations of $c_{I\text{-}N}$ reproduce the experimental trend, that is a decreasing start concentration of LLCPS with increasing pH, the calculated values of $c_{I\text{-}N}$ are significantly underestimated. In particular, while the experimental $c_{I\text{-}N}$ shows a mild dependence on pH (see Figure 4E in the main text), the theoretical calculations show a much stronger pH dependence. Consequently, we note that the condition $D_{eff} \ll L$, required for the validity of the theory (Equations (11)-(14)), is only marginally satisfied at pH 2.0. As a result, comparisons between theory and experiment at higher pH values and using I_{nom} should be considered qualitative at best (*2 , 8-10*).

Table SI 4-1: Theoretical calculations of the start concentration of LLCPS for lysozyme amyloid fibrils ($D = 3.9$ nm, $L = 370$ nm) as a function of pH. Calculations were performed using the nominal ionic strength I_{nom} corresponding to each pH.

pH	λ (e/nm)	I_{nom} (mM)	κ^{-1} (nm)	A'	D_{eff} (nm)	D_{eff}/D	L/D_{eff}	h	Theoretical c_{I-N} (wt %)
2	3.0	10	3.0315	57.1567	18.4993	4.7434	20.0007	0.16387	1.2018
3	2.2	1	9.5863	149.2799	59.2703	15.1975	6.2426	0.16174	0.37513
4	1.6	0.1	30.3146	304.3405	200.5901	51.4334	1.8446	0.15113	0.10992
5	1.4	1.0001×10^{-2}	95.8584	794.8886	717.8872	184.0736	0.5154	0.13353	0.030267
6	1.23	1.01×10^{-3}	301.6417	1981.8334	2526.2055	647.745	0.14646	0.11941	0.0085016
7	1.17	2×10^{-4}	677.8555	4057.9931	6157.8727	1578.9417	0.060086	0.11008	0.0034612

(II) To account for the effect of residual ions (*12*), the ionic strength inside the solution I_{in} was iteratively adjusted until the computed $c_{I\text{-}N}$ agreed with the experimental mean value at each pH (c.f. Figure 4 in the main text). The calculated parameters, including I_{in}, κ^{-1}, A', D_{eff}, and h, are reported in Table SI 4-2. As expected, $I_{in} > I_{nom}$ (compare with Table SI 4-1). We note a reduction in the residual ionic strength $I_o = I_{in} - I_{nom}$ (Equation (17)) with increasing pH, which can arise from

two factors: (a) the lower charge density of the fibrils at higher pH, which partitions fewer counterions, and (b) the longer duration of dialysis at the higher pH values.

Table SI 4-2: Theoretical parameters of LLCPS in solutions of lysozyme amyloid fibrils ($D = 3.9$ nm, $L = 370$ nm), obtained from experimental $c_{\text{I-N}}$ and iterative adjustment of I_{in} at each pH.

pH	λ (e/nm)	Experimental $c_{\text{I-N}}$ (wt %)	Adjusted I_{in} (mM)	κ^{-1} (nm)	A'	D_{eff} (nm)	D_{eff}/D	L/D_{eff}	h
2	3.0	1.49	15.311	2.4499	41.3086	14.9031	3.8213	24.827	0.16439
3	2.2	0.97	5.3703	4.1367	48.4535	23.1387	5.933	15.9905	0.17878
4	1.6	0.90	3.6308	5.031	33.7505	25.4786	6.533	14.522	0.19746
5	1.4	0.83	2.8184	5.7102	30.7404	27.8585	7.1432	13.2814	0.20497
6	1.23	0.68	1.8197	7.1064	31.7739	33.9517	8.7056	10.8978	0.20931
7	1.17	0.58	1.349	8.2538	34.9137	39.5812	10.149	9.3479	0.20853

SI 5. Theoretical Calculations of Pair-Interaction Potential

We considered the interaction between two identical cylindrical colloidal particles of diameter D and length L immersed in an aqueous electrolyte solution. The solvent was treated as a continuum with dielectric permittivity $\varepsilon_r \varepsilon_0$. The ionic strength I was according to Equation (17), and the corresponding Debye screening length κ^{-1} computed from Equation (4). In the following, the equation parameters have the same definition as stated in the main text, and as given in SI 3.

A. Electrostatic interaction potential

The electrostatic interaction between two charged rods were calculated using Debye–Hückel approximation (*13*). Each rod was modeled as a uniformly charged line with linear charge density λ, obtained from the treatments detailed previously in SI 3. For two parallel rods separated by a center-to-center distance x, the electrostatic interaction potential is (*13*):

$$U_{\text{elec}, \parallel}(x) = \frac{2\lambda^2}{\varepsilon_r \varepsilon_0} L K_0(\kappa x) \tag{18}$$

where K_0 is the modified Bessel function of the second kind. For two perpendicular (infinite) rods separated by distance x, the interaction potential is (*13*):

$$U_{\text{elec}, \perp}(x) = \frac{2\pi\lambda^2}{\varepsilon_r \varepsilon_0 \kappa} e^{-\kappa x} \tag{19}$$

These expressions describe the screened electrostatic repulsion between fibrils.

For the calculations (see Figure 7 in the main text), linear charge densities and in-solution ionic strengths according to Table SI 4-2 were used.

B. van der Waals interaction potential

The van der Waals interaction potential between two finite cylindrical colloidal particles was calculated using a compact analytic expression that interpolates near-field and far-field asymptotic regimes and includes finite-size corrections (*14*). For two cylinders separated by a center-to-center distance x, the interaction potential is given by (*14*):

$$U_{\text{vdW}}(x,\theta) = \frac{-A\pi D^4}{32} \frac{\gamma}{\left(|\sin\theta| + 2.35\frac{\sqrt{x(x-\gamma_a D)}}{L}\right)(x-\gamma_a D)(x+0.12D)^3} \tag{20}$$

where A is the Hamaker constant, θ the inter-rod angle ($\theta = 0$ for parallel, $\theta = \pi/2$ for perpendicular), γ and γ_a are finite-length correction factors (see Equation 18 in Ref. (*14*) for additional details). This formula reproduces the parallel and perpendicular asymptotic limits. A Hamaker constant of $3kT$ was assumed (*15*) in the calculations presented in Figure 7 (main text).

C. Total interaction potential

The total pair potential was computed as the sum of the electrostatic and van der Waals contributions:

$$U(x,\theta) = U_{\text{elec}}(x,\theta) + U_{\text{vdW}}(x,\theta) \tag{21}$$

The potentials were evaluated numerically for inter-fibril distances ranging from slightly above contact, $x = 1.05D$. Both parallel ($\theta = 0$) and perpendicular ($\theta = \pi/2$) configurations were analyzed. The results are shown in Figure 7 (main text).

D. Sensitivity analysis

We evaluated the sensitivity of the total interaction potential (and its components) on system parameters including fibril diameter D and linear charge density λ, in-solution ionic strength I_{in} , and the Hamaker's constant A .

Fibril diameter. The fibril height at each pH was measured using AFM (see Figure 1F in the main text). For the calculation of the interaction potential presented in Figure 7 in the main text, the fibril diameter was estimated as the mean of the statistical average fibril height H at each pH, resulting in $D = 3.9$ nm . To evaluate the sensitivity of the calculations to this parameter, the interaction potential was recalculated considering a 50% confidence interval, that is, for $D = 2.0$ nm and 5.9 nm. The results are presented in Figure SI 5-1.

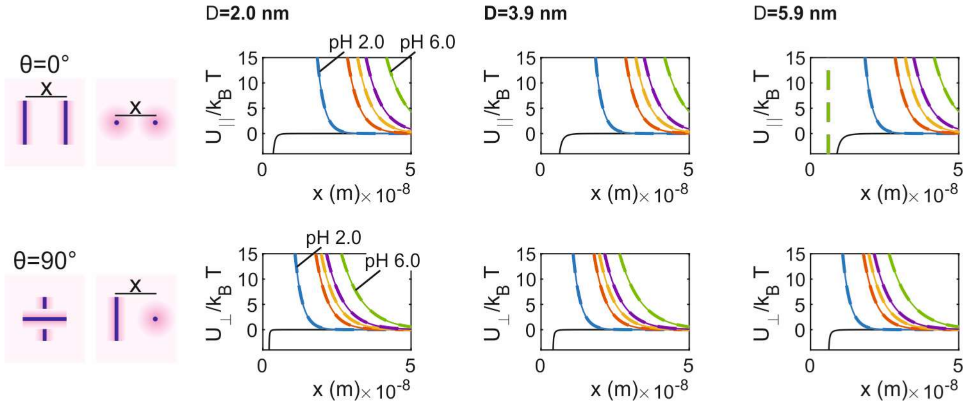

Figure SI 5-1: Interaction potential between two lysozyme amyloid fibrils aligned in parallel (θ=0°) or perpendicularly (θ=90°) as a function of distance x, for pH 2.0 to 6.0. The interaction potential was calculated for fibril diameters ranging 2.0 to 5.9 nm as indicated on the figure panels. Solid colored line: electrostatic interaction; black solid line: van der Waals (vdW) interaction; dashed colored line: total interaction potential.

As expected, there is a noticeable variation in the van der Waals interaction potential with fibril diameter. In particular, in both parallel and perpendicular geometries, with increasing fibril diameter, the van der Waals interaction potential increases. Nevertheless, for an assumed Hamaker constant of $3kT$ (*15*), the total interaction potential is dominated by the electrostatic interaction potential at pH 2.0 to 6.0.

Fibril linear charge density. In **SI 3** and in Methods (main text) we described the experimental and theoretical methodology to obtain the linear charge density at each pH value. In Table SI 1-1 the linear charge density of lysozyme amyloid fibrils were reported. To evaluate sensitivity to calculations of the linear charge density, we varied this parameter to the extremes of its 95% confidence intervals (see Table SI 5-1) and recalculated the interaction potentials at the lower/higher intervals as well as the best fit values. The results are presented in Figure SI 5-2.

Table SI 5-1: The best fit value and the lower and upper 95% confidence intervals in linear charge density of lysozyme amyloid fibrils extracted from Table SI 1-1.

pH	Best fit λ (e/nm)	Interval (e/nm)
2.0	3.0	[2.8 3.2]
3.0	2.2	[2.1 2.3]
4.0	1.6	[1.4 1.7]
5.0	1.4	[1.2 1.5]
6.0	1.23	[1.07 1.39]

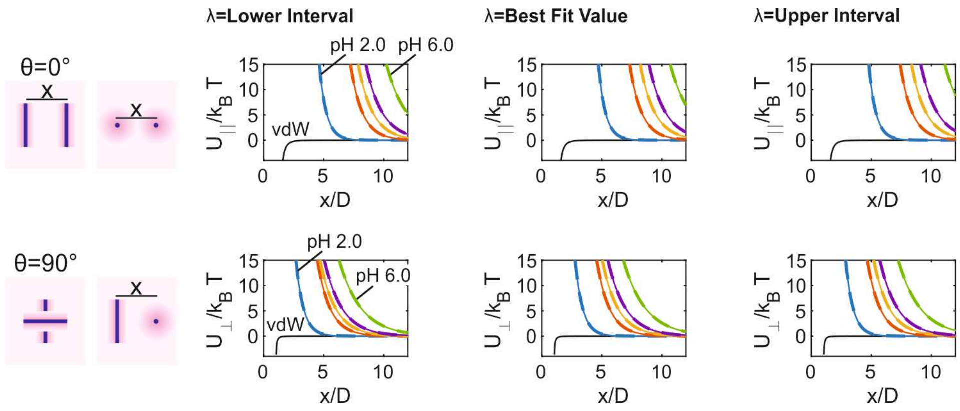

Figure SI 5-2: Interaction potential between two lysozyme amyloid fibrils aligned in parallel (θ=0°) or perpendicularly (θ=90°) as a function of the normalized distance x/D, for pH 2.0 to 6.0. The interaction potential was calculated for fibril linear charge densities reported in Table SI 5-1. Solid colored line: electrostatic interaction; black solid line: van der Waals (vdW) interaction; dashed colored line: total interaction potential.

There is a noticeable increase in the electrostatic interaction potential with fibril linear charge density in parallel geometry; however, these variations are less noticeable in perpendicular geometry. The total interaction potential is dominated by the electrostatic interaction potential at lower/higher as well as the best fit values of the linear charge densities.

In-solution ionic strength. In Table SI 4-2 the in-solution ionic strengths of lysozyme amyloid fibrils adjusted to match the experimental start of LLCPS (see Figure 4 in the main text and SI 4) were reported. To evaluate sensitivity to its values at each pH, we varied this parameter to 50% intervals (Table SI 5-2) and recalculated the interaction potentials at the lower/higher intervals. The results are presented in Figure SI 5-3.

Table SI 5-2: The in-solution ionic strength of lysozyme amyloid fibrils extracted from Table SI 4-2, and its 50% lower and upper intervals.

pH	In-solution I_o (mM) (from experimental LLCPS)	Interval (mM)
2.0	15.3	[7.7 23.0]
3.0	5.4	[2.7 8.1]
4.0	3.6	[1.8 5.4]
5.0	2.8	[1.4 4.2]
6.0	1.8	[0.9 2.7]

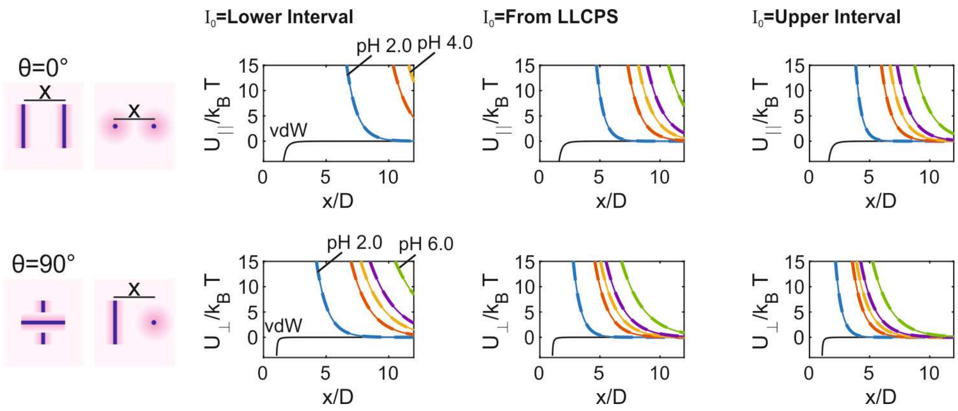

Figure SI 5-3: Interaction potential between two lysozyme amyloid fibrils aligned in parallel (θ=0°) or perpendicularly (θ=90°) as a function of the normalized distance x/D, for pH 2.0 to 6.0. The interaction potential was calculated for in-solution ionic strength reported in Table SI 5-2. Solid colored line: electrostatic interaction; black solid line: van der Waals (vdW) interaction; dashed colored line: total interaction potential.

There is a significant decrease in the electrostatic interaction potential with in-solution ionic strength in both parallel and perpendicular geometries. Although the total interaction potential is dominated by the electrostatic interaction potential at these linear charge densities, this result shows the important effect of ionic strength on LLCPS of amyloid fibrils, and transition to LLPS.

Hamaker constant. We are not aware of a measurement of amyloid fibril Hamaker constant; however, Hamaker constant of typical proteins have been reported to be about $3kT$ (*15*). We employed this value in the default calculations presented in the main text (Figure 7), and below present new calculations considering a 50% uncertainty it its value, that are at $1.5kT$ and $4.5kT$, including a very high estimation at $100kT$.

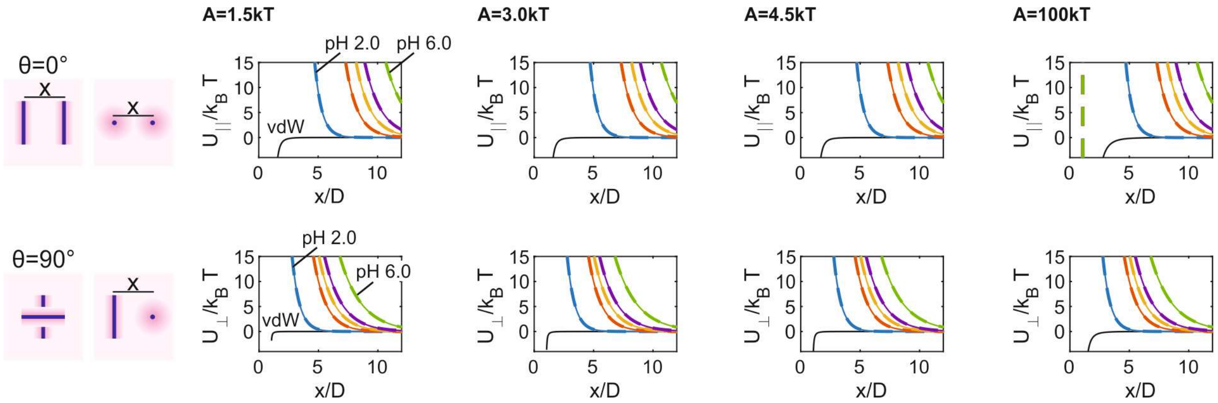

Figure SI 5-4: Interaction potential between two lysozyme amyloid fibrils aligned in parallel (θ=0°) or perpendicularly (θ=90°) as a function of the normalized distance x/D, for pH 2.0 to 6.0. The interaction potential was calculated for various values of the Hamaker constant reported on top of each panel. Solid colored line: electrostatic interaction; black solid line: van der Waals (vdW) interaction; dashed colored line: total interaction potential.

We find that within 50% uncertainty interval, the variations in van der Waals interaction potential with Hamaker constant are minute; although, as expected, the van der Waals interaction potential increases with Hamaker constant most noticeably at a high value of $100kT$ (*15*).

SI 6. SI Methods

Inductively coupled plasma mass spectrometry (ICP-MS). The concentrations of cationic species, including Na^{+}, Mg^{2+}, K^{+}, and Ca^{2+}, were determined using ICP-MS (iCap RQ, Thermo Fisher Scientific). For each measurement, 250 µl of lysozyme amyloid fibril (Lys-B) solutions at pH 2.0, 3.0, 4.0, 5.0, and 6.0, with concentrations of 2.0, 1.4, 1.6, 1.6, and 1.2 wt %, respectively, were analyzed in triplicate. Prior to analysis, 1 ml concentrated HNO_3 was added to each sample to achieve mineralization, and the samples were treated using a microwave autoclave (TurboWave, MLS) at 250°C for 30 min. Following this step, the samples were diluted with Milli-Q water to a final volume of 15 mL before ICP-MS measurement.

ThT assay. Thioflavin T (ThT, Sigma-Aldrich) fluorescence of lysozyme amyloid fibril at pH 2.0 to 8.0 was performed as a function of fibril concentration in the dilute single fibril phase. ThT stock solution (20 mM in DMSO) was diluted a factor of twenty in the respective pH-adjusted Milli-Q water and filtered using 0.2 µm PTFE syringe filters. Then after, 10 µl was added to 190 µl of amyloid solution. The fluorescence intensity was measured with an excitation wavelength and emission wavelength of 440 nm and 480 nm, respectively.

Circular dichroism (CD). Lysozyme amyloid fibril solutions at pH 2.0 to 8.0 were diluted to 0.025 wt % using respective pH-adjusted Milli-Q water and loaded into a high-quality quartz cuvette with 1 mm optical path length (Postmann Instruments). CD spectra were collected using a Jasco J-815 CD spectrometer with a wavelength ranging from 190–260 nm, data pitch of 0.2 nm, and scanning speed of 20 nm/min. Five consecutive measurements were performed.

References

1. I. Usov, R. Mezzenga, FiberApp: An open-source software for tracking and analyzing polymers, filaments, biomacromolecules, and fibrous objects. *Macromolecules* **48**, 1269-1280 (2015), https://doi.org/10.1021/ma502264c.
2. P. Azzari, R. Mezzenga, LLPS vs. LLCPS: analogies and differences. *Soft Matter* **19**, 1873-1881 (2023), https://doi.org/10.1039/D2SM01455F.
3. H. Almohammadi, S. A. Khadem, P. Azzari, Y. Yuan, A. Guerra, A. D. Rey, R. Mezzenga, Liquid–liquid crystalline phase separation of filamentous colloids and semiflexible polymers: experiments, theory and simulations. *Reports on Progress in Physics* **88**, 036601 (2025), https://doi.org/10.1088/1361-6633/adb441.
4. H. Ohshima, Henry's function for electrophoresis of a cylindrical colloidal particle. *Journal of Colloid and Interface Science* **180**, 299-301 (1996), https://doi.org/10.1006/jcis.1996.0305.
5. J. N. Israelachvili, *Intermolecular and Surface Forces* (Academic Press, Boston, ed. Third Edition, 2011).
6. H. Ohshima, Surface charge density/surface potential relationship for a cylindrical particle in an electrolyte solution. *Journal of Colloid and Interface Science* **200**, 291-297 (1998), https://doi.org/10.1006/jcis.1998.5433.
7. J. Adamcik, J.-M. Jung, J. Flakowski, P. De Los Rios, G. Dietler, R. Mezzenga, Understanding amyloid aggregation by statistical analysis of atomic force microscopy images. *Nature Nanotechnology* **5**, 423-428 (2010), https://doi.org/10.1038/nnano.2010.59.
8. L. Onsager, The effects of shape on the interaction of colloidal particles. *Annals of the New York Academy of Sciences* **51**, 627-659 (1949), https://doi.org/10.1111/j.1749-6632.1949.tb27296.x.
9. G. J. Vroege, H. N. W. Lekkerkerker, Phase transitions in lyotropic colloidal and polymer liquid crystals. *Reports on Progress in Physics* **55**, 1241 (1992), https://doi.org/10.1088/0034-4885/55/8/003.
10. A. Stroobants, H. N. W. Lekkerkerker, T. Odijk, Effect of electrostatic interaction on the liquid crystal phase transition in solutions of rodlike polyelectrolytes. *Macromolecules* **19**, 2232-2238 (1986), https://doi.org/10.1021/ma00162a020.
11. Y. Cao, S. Bolisetty, G. Wolfisberg, J. Adamcik, R. Mezzenga, Amyloid fibril-directed synthesis of silica core–shell nanofilaments, gels, and aerogels. *Proceedings of the National Academy of Sciences* **116**, 4012-4017 (2019), https://doi.org/10.1073/pnas.1819640116.
12. F. G. Donnan, The theory of membrane equilibria. *Chemical Reviews* **1**, 73-90 (1924), https://doi.org/10.1021/cr60001a003.
13. S. L. Brenner, V. A. Parsegian, A physical method for deriving the electrostatic interaction between rod-like polyions at all mutual angles. *Biophysical Journal* **14**, 327-334 (1974), https://doi.org/10.1016/S0006-3495(74)85919-9.
14. J. A. Logan, A. V. Tkachenko, Compact interaction potential for van der Waals nanorods. *Physical Review E* **98**, 032609 (2018), https://doi.org/10.1103/PhysRevE.98.032609.
15. C. M. Roth, B. L. Neal, A. M. Lenhoff, Van der Waals interactions involving proteins. *Biophysical Journal* **70**, 977-987 (1996), https://doi.org/10.1016/S0006-3495(96)79641-8.
